# Super-resolution tactile sensor arrays with sparse units enabled by deep learning

**DOI:** 10.1126/sciadv.adv2124

**Published:** 2025-07-02

**Authors:** Depeng Kong, Yuyao Lu, Shuyao Zhou, Mengke Wang, Gaoyang Pang, Baocheng Wang, Lipeng Chen, Xiaoyan Huang, Honghao Lyu, Kaichen Xu, Geng Yang

**Affiliations:** ^1^ State Key Laboratory of Fluid Power and Mechatronic Systems, School of Mechanical Engineering, Zhejiang University, Hangzhou 310030, China.; ^2^ School of Electrical and Computer Engineering, The University of Sydney, Sydney 2006, Australia.; ^3^ Tencent Robotics X Laboratory, Tencent, Shenzhen 518057, China.; ^4^ College of Electrical Engineering, Zhejiang University, Hangzhou 310027, China.; ^5^ Zhejiang Key Laboratory of Intelligent Robot for Operation and Maintenance, Hangzhou 310030, China.

## Abstract

High-resolution tactile perception is essential for humanoid robots to perform contact-based interaction tasks. However, enhancing resolution is typically accompanied by increasing the density of sensing nodes, large numbers of interconnecting wires, and complex signal processing modules. This work presents super-resolution (SR) tactile sensor arrays with sparsely distributed taxels powered by a universal intelligent framework. Such smart sensor systems involve a general topological optimization strategy for taxel layout design and a deep learning model called self-attention–assisted tactile SR. Driven by the proposed model, they can dynamically distinguish high-density pressure stimuli by generating 2700 virtual taxels from only 23 physical taxels. An SR scale factor of more than 115 and an average localization error of 0.73 millimeters are achieved, approximating human fingertip accuracy and surpassing current state-of-the-art solutions. This framework enhances flexible sensors with SR capabilities in a facile and energy-efficient manner, illustrating the potential to equip robots with embodied tactile perceptions.

## Teaser

A tactile super-resolution framework boosts robotic touch accuracy, enabling human-like precision with fewer sensors.

## INTRODUCTION

Embodied perception is fundamental to embodied artificial intelligence (*1*), allowing humanoid robots to comprehend their environment and human intentions, thus enabling complex and high-level tasks (*2*, *3*). Despite visual perception, it is essential to incorporate tactile sensation to overcome vision limitations (*4*), particularly when visual information is insufficient, ambiguous, or entirely unavailable, such as in unstructured environments and dark or occluded spaces (*5*, *6*). Tactile sensors are typically array-like with a grid of sensing units (taxels). Their mechanisms range from resistive (*7*–*10*), capacitive (*11*–*13*), triboelectric (*14*–*16*) to magnetic (*17*, *18*) working principles. Higher spatial resolution is essential to capture finer haptic details and enable precise manipulation. However, enhancing resolution at the physical level is typically performed by designing diverse taxels, with many interconnecting wires in a densely packed configuration. This brings challenges related to fabrication, reliability, signal cross-talk, and the efficient processing of massive tactile data that are continuously generated.

**Fig. 1.**
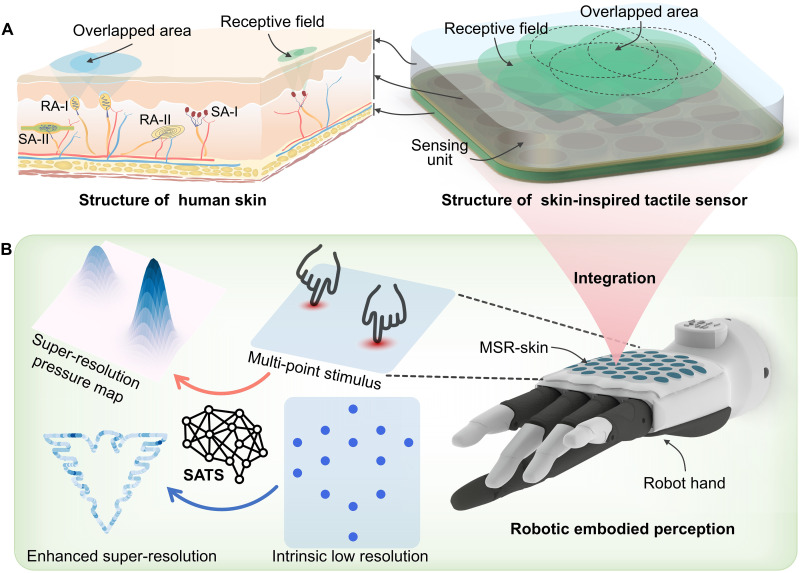
Skin-inspired design and SR sensing capability of the tactile sensor. (**A**) Schematic illustration of the structure of human skin and the skin-inspired tactile sensor. Both structures have an elastic layer as the transmission medium between external stimuli and sensing units with overlapped receptive fields. SA and RA indicate slowly adapting and rapid adapting, respectively. (**B**) Illustration of the tactile sensor integrated into a robot hand. The designed tactile SR system is capable of both single-point and multi-point perception while extensively boosting intrinsic spatial resolution.

Although vision-based tactile sensors afford a solution to high-resolution perception using cameras and vision-based algorithms (*19*, *20*), they are often constrained by limited structural design and large size, which restricts their scalability across various robotic tactile sensing requirements. In nature, human skin serves as inspiration to address such issues, where mechanoreceptors (MRs) (*21*, *22*) detect stimuli within a surrounding area known as the receptive field (*23*, *24*). Human skin can perceive stimuli with a spatial resolution finer than the physical resolution of MRs, a phenomenon called tactile super-resolution (SR) (*25*, *26*). This ability is facilitated by the overlap of receptive fields between adjacent MRs (Fig. 1A), which allows stimulus localization by synthesizing signals from multiple sources (*26*, *27*). In this process, the nervous system functions as a signal processor, using a minimal number of sensors to decode external stimuli accurately. Deep learning models can effectively replicate this capability by learning and integrating multisource signals, a concept extensively explored in existing literature (*28*–*30*). Building on the mechanism of human skin’s tactile SR, several tactile sensors have achieved SR using deep learning techniques for either single-point (*31*–*35*) or multi-point contact sensing (summarized in table S1) (*36*–*39*). Multi-point contact sensing is preferred due to its universality in real-world applications. However, acquiring large amounts of data for deep model training across diverse contact scenarios remains challenging (*40*, *41*). Researchers have explored solutions from the signal perspective, using magnetized film and Hall sensors to decouple multi-point presses into several single-point presses (*42*, *43*). Multi-point contact signals were synthesized by manually stacking multiple single-point presses, which were then used to train a deep model for multi-point contact recognition. While this approach effectively reduces the demand for massive data collection, it still limits the trained model by the diversity of the training data.

While simulation methods and knowledge transfer techniques have provided some solutions (*36*–*39*), the domain shift between simulated and real-world data reduces perceptual accuracy. Furthermore, achieving high-resolution sensation with minimal taxels is crucial for large-area deployment. This requires optimizing the use of available sensing resources by fully using the receptive fields of the taxels. However, current research has not yet addressed optimizing taxel arrangements based on their receptive fields, leading to redundancy and wasted resources.

This paper proposes a universal computational framework that leverages deep learning techniques to endow tactile sensors with the perception of SR pressure distribution. A general topological method is proposed to optimize the layout design of the tactile sensor array, which ensures the most efficient utilization of limited sensing units, contributing to using the fewest units to cover the largest area. Building on this layout, an end-to-end self-attention–assisted tactile SR (SATS) framework is designed to estimate the distribution of stimuli (e.g., pressure) across the sensing surface by processing multichannel signals. A flexible 23-node tactile sensor was fabricated and calibrated by the SATS model to compose a multi-point SR skin (MSR-skin) to validate this framework. Two thousand seven hundred virtual taxels were generated from 23 physical taxels, and an SR scale factor (defined in note S1) of ~117 was achieved. The average localization error across the sensing surface was 0.73 mm [root mean square error (RMSE)], which is similar to the human fingertip’s localization accuracy (0.3 to 1 mm) (*25*, *44*) and superior to state-of-the-art approaches. As far as we know, this is the first solution to multi-point tactile SR for flexible sensors that can perceive the pressure distribution across the sensing surface.

The MSR-skin was integrated into a robot hand, endowing it with embodied tactile perception (Fig. 1B). Besides, to intuitively demonstrate the SR capability, a soft keyboard with a highly compact layout was integrated into the sensing surface as an input device for various applications of human-machine interaction. Moreover, under its contact map–oriented perception with SR, the sensor successfully imaged various shapes and accurately recognized them, achieving a classification accuracy of 98.35%. The generality of the proposed SR framework was further demonstrated by guiding the design of a dynamic SR tactile sensor based on triboelectric nanogenerators (TENGs).

## RESULTS

### Skin-inspired design and layout optimization

**Fig. 2.**
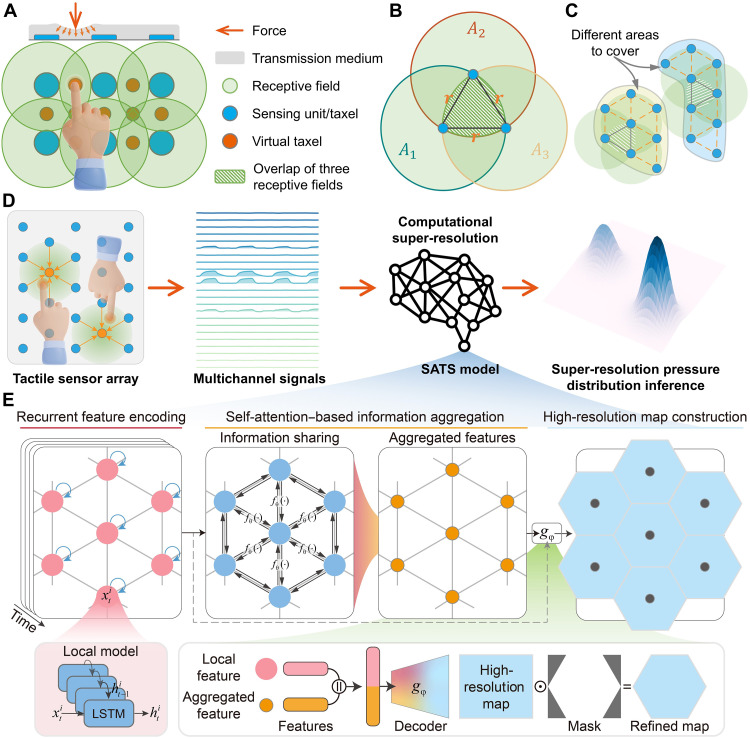
The proposed tactile SR framework. (**A**) Principle of generating virtual taxels by leveraging overlapped receptive fields. (**B**) Optimized taxel layout that maximizes the area of receptive fields. (**C**) Coverage of two arbitrarily shaped areas through the extension of new taxels. The yellow and blue shaded areas represent the regions to be covered, while the black and dotted lines denote edges connecting adjacent sensing units after constructing the graph representation. The blue dots represent the sensing units, and the green circles indicate their respective receptive fields. (**D**) Schematic illustration of the SR sensing process, where multichannel signals are monitored from the tactile sensor array and processed by the SATS model to infer the pressure distribution. (**E**) The proposed SATS model, including recursive feature encoding, self-attention–based information aggregation, and high-resolution map construction. Each node in this seven-node graph represents a taxel’s feature vector encoded by a corresponding module at a certain phase.

Drawing inspiration from the mechanism of human skin’s tactile SR, we propose a general framework for artificial tactile SR. As shown in Fig. 1A, human skin has a multilayered structure, primarily composed of the epidermis, dermis, and subcutaneous tissue. MRs, responsible for tactile perception, are mainly located within the dermis (*21*). The epidermis primarily comprises keratinocytes, which protect deeper skin structures and the whole body. The dermis, rich in collagen and elastic fibers, provides elasticity while offering mechanical support to the embedded MRs. Beneath the dermis, the subcutaneous tissue comprises an adipose layer and loose connective tissue, functioning as supportive and cushioning structures. When an external force is applied to the skin, the epidermis and dermis spread the mechanical stimulus over a broader area, activating several MRs simultaneously. This enables the somatosensory nervous system to synthesize signals from multiple MRs, thereby enhancing the accuracy of force localization. In other words, the epidermis and dermis enlarge the receptive fields of MRs and create overlapped areas of them, forming the basis for tactile SR. The structure of a tactile sensor array is analogous to human skin, which includes sensing units and a transmission medium that extends the receptive field of each sensing unit. For instance, an elastic cover can spread the external force over a wider area, allowing for stimulus detection in regions without sensing units (Fig. 2A). By synthesizing data from activated sensing units, virtual taxels are generated between physical taxels, substantially improving the tactile sensor’s spatial resolution.

An overlapped receptive field is essential for achieving tactile SR, necessitating the investigation of the most efficient array layout to maximize the use of available sensing resources. On a two-dimensional plane, at least three noncollinear points are required to determine the position of any other point based on its relative positions to three known points (*45*). By analogy, an applied force, depending on its magnitude and the distance from a taxel, induces different responses from the taxel. The precise location and magnitude of the contact force can be inferred by analyzing responses from at least three noncollinear taxels along with their locations.

Consequently, three taxels serve as the fundamental unit for optimizing array layout (fig. S1A). This optimization problem can be formulated as

$$\begin{array}{c} d_{1}^{*},d_{2}^{*},d_{3}^{*}=\text{arg}\underset{d_{1},d_{2},d_{3}}{\text{max}}\left(A_{1}\cup A_{2}\cup A_{3}\right),\;subject\;to\;d_{1}\leq r,d_{2}\leq r,d_{3}\leq r \end{array}$$ (1)

where *d*_1_, *d*_2_, and *d*_3_ represent the distances between taxels, *r* denotes the radius of the receptive field, and *A*_1_ = *A*_2_ = *A*_3_ = π*r*^2^ corresponds to the area of each taxel’s receptive field. By adjusting *d*_1_, *d*_2_, and *d*_3_, the goal is to maximize the union area of these receptive fields while ensuring that all three receptive fields fully cover the triangular area formed by the three taxels. In other words, by solving this problem and maximizing the union area of the three units, the primary goal of tactile SR, namely, using the fewest taxels to cover the largest area, can be achieved. The particle swarm optimization (PSO) algorithm was used to solve this problem, and the optimized layout was achieved with $d_{1}^{*}=d_{2}^{*}=d_{3}^{*}=r$ . (Fig. 2B and fig. S1, B and C). On the basis of this fundamental layout, new taxels could be extended following the same pattern to cover surfaces with arbitrary shapes (Fig. 2C). To be noted, the PSO algorithm was chosen to solve this problem due to its fast convergence and superior performance. Other machine learning methods could also be applied, and a comparison of several optimization algorithms is provided in note S2 and fig. S2. In addition, it is assumed that the three receptive fields have the same radius in the current setup. However, the optimization method can be extended to handle cases where the receptive fields have different radii. Further details are provided in note S2 and fig. S3.

### The SATS deep learning model

A tactile sensor array organized in the optimized layout can be conceptualized as a graph, where the nodes represent sensing units and the edges connect adjacent nodes, determined by the receptive fields, as shown in Fig. 2C. An SATS model, tailored to this structure, is proposed to process the original signals from the sensing units and estimate the pressure distribution across the entire sensing surface (Fig. 2D). The SATS model comprises four components: a long short-term memory (LSTM)–based recurrent feature encoding module, a self-attention–based information aggregation module, a local map construction module, and a convolutional neural network (CNN)–based refining module, as depicted in Fig. 2E.

LSTM networks are well-suited for encoding time-series data and have proven effective in modeling the hysteresis inherent in elastic sensing materials (*46*). Given that sensing units, even those from the same batch, have distinct response characteristics, a unique LSTM encoder is assigned to each sensing unit to accommodate its specific properties. The features encoded in this manner, called local features, cannot capture spatial touch information since a single point cannot define a plane. Therefore, a self-attention module, which has been proven effective in aggregating information from related parts (*47*), is introduced to facilitate information sharing among adjacent sensing units. This module learns to aggregate information from neighbor units into the current unit, thereby integrating multisource data to infer spatial touch information (Fig. 2E, middle). It allows for consideration of both the primary responsiveness of each sensing unit and the cooperation among adjacent sensing units. Besides, this approach naturally integrates the domain knowledge of the tactile SR mechanism, where the stimulus information on the sensing surface can be inferred by analyzing the responses of at least three sensing units.

Figure 2E (middle) illustrates the information aggregation process from six adjacent units to one central unit, with details provided in note S3. The features aggregated through this process were considered to contain stimulus information surrounding the central unit. These aggregated features are then concatenated with the original local features and fed into a multilayer perceptron (MLP)–based decoder (function $g_{\varphi }$ ) to construct local maps for each unit individually. All local maps are merged, according to the location of sensing units, to form the overall map. In this condition, each sensing unit is primarily responsible for constructing its own local map and partially contributes to the local maps of its neighboring units. When multiple contacts simultaneously stimulate areas of different local maps, this partitioning strategy enables local map inference in a relatively independent and parallel manner. This is analogous to decomposing a multi-point contact into several single-point contacts. From the perspective of deep models, the SATS mode extracts local features and constructs local maps. This design enables the local networks, including the LSTMs for each sensing unit and the MLP for local map construction, to focus on stimuli near a specific sensing unit and construct corresponding local maps. In this manner, the local networks solve nearly identical problems for both single-point and multi-point presses, decomposing a multi-point contact into several single-point contacts while maintaining a consistent data distribution for the local networks. This allows the sensing system to be directly applied to multi-point contact scenarios despite being calibrated with only single-point contact data. It effectively mitigates the domain shift issue (*48*) between single-point and multi-point contact data, substantially enhancing its generality to real-world applications. Last, two convolutional layers were used to refine the integrated map, providing more substantial fitting capabilities and smoother expressions to merge local pressure maps from individual taxels more effectively.

Through the above procedures, the SATS model takes raw multichannel signals from the sensor array as input and outputs a two-dimensional matrix representing the inferred pressure distribution across the sensing surface. This matrix has a size of 54 by 50, with each value corresponding to the pressure at a specific position on the sensing surface, which is considered a virtual taxel. Therefore, the SATS model generates 54 by 50 = 2700 virtual taxels in a single inference. The structural design of the SATS model incorporates both the general signal characteristics of tactile sensors and the inherent structure of sensor arrays, ensuring computational efficiency and broad applicability across various scenarios. This distinguishes SATS from existing approaches that use general machine learning models without explicitly considering SR scenarios, such as plain MLPs (*8*, *17*, *49*), interpolation models (*42*, *43*), and CNNs (*39*). While these methods enable SR tactile sensing, they heavily rely on high-quality annotated data for model training. This requirement becomes even more demanding in multi-point SR tasks. In contrast, the proposed SATS model demonstrates superior learning efficiency by leveraging knowledge transfer from single-point to multi-point contact in a zero-shot manner.

### Fabrication and characterization of the soft tactile sensor

**Fig. 3.**
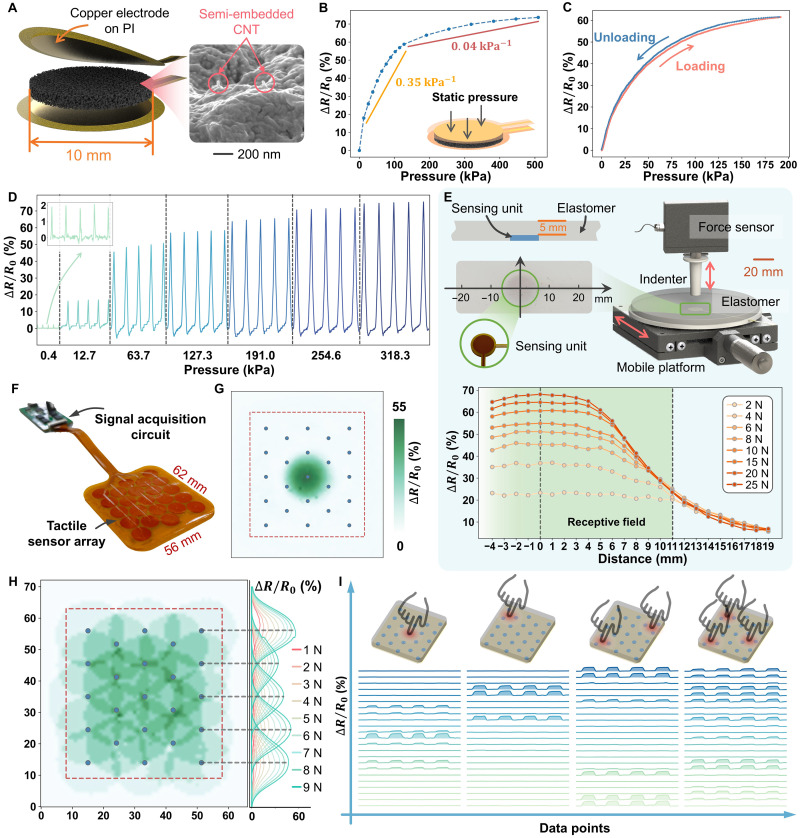
Fabrication and characterization of the tactile sensor. (**A**) Explosive view of the fabricated sensing unit and SEM image showing the material’s surface embedded with CNTs. PI, polyimide. (**B**) Relative resistance changes under varying pressures, where $R_{0}$ represents resistance without applied pressure. (**C**) Hysteresis of the sensing unit. (**D**) Dynamic response under pressures ranging from 12.7 to 318.3 kPa. (**E**) Setup for measuring the receptive field of a single sensing unit and the corresponding results. (**F**) Photograph of the sensor array prototype. (**G**) Receptive field of the central sensing unit calibrated under an external force of 8 N. Blue dots indicate real taxels. (**H**) Overall receptive field of the tactile sensor, obtained by stacking the receptive fields of all taxels. The right section view shows the responses of the five rightmost taxels under various forces. (**I**) Response characteristics of the sensor array under pressure applied at one, two, and three points, respectively.

A Piezoresistive material was selected to fabricate the sensing units for stability and durability. As shown in fig. S4, the porous carbon nanotube (CNT)–based sponge was manufactured through a typical acid etching process using nickel (Ni) foams with a porosity of 110 pixels per inch (PPI) as the porous template. First, a mixture of CNT and chloroform was prepared and sonicated using an ultrasonic cell disruption system. Next, the mix of polydimethylsiloxane (PDMS) precursor and curing agent was diluted by adding chloroform. The diluted PDMS precursor solution was added to the CNT/chloroform mixture and stirred at room temperature. The resulting mixture (CNT/chloroform/PDMS) was then heated and stirred to evaporate a portion of the chloroform. Ni foams were cut into circular shapes using an infrared laser system. These Ni foams were briefly immersed into the CNT/chloroform/PDMS mixture and shaken on a sieve to remove uneven or cohesive parts. After torrefying, the cured samples were immersed in a diluted hydrochloride acid (HCl) solution for etching. Last, the porous CNT/PDMS sponges were obtained by washing with distilled water for several times. Figure 3A demonstrates the fabricated unit with a diameter of 10 mm and a thickness of 2 mm. The porous structure of the sensing material can be observed by a scanning electron microscope (SEM) (fig. S5). The conductivity of this material was provided by the CNTs semi-embedded in the PDMS. Conductivity changes under pressure due to the contact and separation of these CNTs.

A sensing unit was fabricated to characterize the sensing material by attaching two electrodes to the top and bottom of the unit using silver paste as the conductive adhesive. The relative resistance change of the sensing unit across its detection range is depicted in Fig. 3B. Under a transient stimulus of 5 N, the sensing unit exhibited a fast response time of 30 ms (fig. S6A). In addition, the hysteresis of the sensing unit was tested (Fig. 3C), where a relatively small range was observed (1.98%). The LSTM can model such slightly different responses during the loading and unloading processes in the SATS model. Furthermore, this sensing unit demonstrated stability and repeatability under dynamic loading (Fig. 3D). The relative resistance change increased incrementally as the external pressure rose from 0.4 to 318 kPa. Notably, this sensing porous was sensitive, capable of detecting pressure as slight as 0.4 kPa (inset of Fig. 3D). To validate the device’s durability, more than 3000 repeated loading-unloading cycles were conducted (fig. S6B), illustrating the durability of the sensing unit as part of a tactile sensor array.

In addition, the performance of CNT/PDMS fabricated by three types of Ni foams with diverse porosities was further studied, as shown in fig. S7. Obviously, the samples with porosity of 80, 110, and 130 PPI were observed with similar sensitivity at pressures below 80 kPa. The CNT/PDMS with the lower porosity and larger pore size shows slightly higher sensitivity at low pressures. However, the 80 and 130 PPI samples show lower sensitivity than the 110 PPI sample at a higher pressure range (100 to 300 kPa). Considering the overall sensitivity performance across a broad pressure range, this study chose the Ni foam with a porosity of 110 PPI for preparing CNT/PDMS piezoresistive sensors. Notably, the Ni foam was used as an acid etching template for preparing porous conductive elastomer instead of compressing material due to its limited elastic deformation and high mechanical strength. Comparisons between pure Ni foam and the fabricated CNT/PDMS sponges are shown in fig. S8.

An elastomer with a thickness of 5 mm was placed on top of the sensing unit to enlarge the receptive field. This rubber-based elastomer effectively distributed the influence of an external force applied on top, as described by contact mechanics (*50*). Figure 3E shows the setup for testing the receptive field of a sensing unit covered by the elastomer. The integrated sensing unit was placed on a mobile platform, allowing the indenter to press each position along its symmetric line by moving the platform. The response of the sensing unit was recorded as different positions were pressed with forces ranging from 2 to 25 N. The sensing unit was capable of detecting an external force at the position 19 mm away from its center. To ensure an evident and distinguishable signal and a decent signal-to-noise ratio, 10.5 mm was lastly determined as the radius of the receptive field, which was then used as the distance between adjacent units in a sensor array.

Figure S9 illustrates the fabrication process of a 23-node sensor array prototype, which serves as the hardware base of the MSR-skin. Initially, the electrode layer, comprising 23 copper electrodes on a polyimide film substrate, was fabricated. A polyethylene terephthalate (PET) film, functioning as a mask, was used to cover the electrode layer and was precisely cut using a digital laser to expose the electrodes. The silver paste was blade-coated onto the electrodes as a conductive adhesive, establishing the connection between the electrodes and sensing units. Taxels at the edges have fewer neighboring taxels than those in central positions, which may result in distinct force conditions and instability in electrode connections. Therefore, a spacer was placed around the perimeter of the sensor array for additional support. Last, a top electrode layer was attached, completing the fabrication process. The 23-node sensor array was obtained (Fig. 3F). A circuit board was designed and fabricated to measure the 23-channel resistance signals from the sensor array using the voltage divider method. The schematic diagram of the circuit is shown in fig. S10.

To evaluate the performance of the sensor array, a grid of positions on the sensing surface was stimulated to obtain the sensor array’s responses. A robot arm with a force sensor at its end was programmed to drive an indenter to press positions on the sensing surface (fig. S11). The robot arm was preprogrammed to move 1 mm per step, partitioning the entire sensing surface (72 mm by 66 mm) into a 72 by 66 matrix, resulting in 4891 pressing points (73 by 67). At each point, it incrementally increased the indentation force up to 10 N using a force feedback control policy. The location of the indenter, measurements from the force sensor, and responses from the sensor array were recorded throughout the data collection process.

On the basis of the acquired data, the receptive field of each taxel was characterized. Specifically, we recorded each taxel’s response (relative resistance change) when pressing each position on the sensing surface with an external force of 8 N. The results are shown in Fig. 3G and fig. 12. For clearer illustration, the receptive fields were filtered by eliminating values below 5% and normalizing the remaining values to 1. The results are shown in fig. S13. Each taxel’s receptive field covers the positions of its neighboring units, which fulfills the design objective of overlapped receptive fields and lays the foundation for subsequent SR implementation. By overlapping the receptive fields of all taxels, the entire receptive field of the sensor array can be obtained, as shown in Fig. 3H. In addition, the responses of the five taxels located on the right side of the sensor array are visualized in Fig. 3H (right). It demonstrates the responses when pressing positions along the common symmetry line of these taxels. When pressing the center of one taxel, its adjacent taxels can also detect this pressure, indicating that their receptive fields encompass this position.

Figure 3I demonstrates the relative resistance change across 23 channels (sensing units) when pressure was applied to one, two, or three points on the sensor array. Different contact patterns yielded distinct responses across 23 channels. These time-series response features allowed the SATS model to learn and infer the pressure distribution over the entire sensing surface with finer details, achieving tactile SR. This characterization illustrates the potential of a tactile sensing system composed of a sensor array for generating high-quality sensing signals and an SATS model for processing and interpreting these signals.

### Calibration and validation of the MSR-skin system

Ground truth, i.e., the expected pressure distribution, is essential for calibrating the MSR-skin comprising the sensor array and the SATS model. Conventionally, two approaches can be used to generate ground truth: direct measurement and simulation. However, measuring the pressure distribution across a surface directly is nearly impossible, and conventional simulation methods, such as the finite element model (FEM), are computationally expensive. To address this issue, a simulation method was proposed to approximate real scenarios based on the elastic half-space (EHS) model, using data recorded by the robot arm (coordinates and force). The EHS model describes how stress evolves inside an elastomer when its top surface is subjected to a point-contact pressure and has been used to model the mechanical properties of tactile sensors (*51*, *52*). A simulation model based on this theory was constructed, as shown in fig. S14A. When a point on the elastomer’s surface is pressed, the stress at another point inside the elastomer is inversely proportional to the distance between them, which can be formulated as detailed in note S4. The pressure distribution was examined when a point load was applied. Figure S14 (B and C) illustrates the pressure distribution for varying external forces and elastomer thicknesses. It is observed that the elastomer’s thickness primarily influences the receptive field. A thicker elastomer leads to a larger receptive field by smoothing the pressure distribution. For a given thickness, a larger external force results in a higher pressure at the same location, thereby enhancing the sensor’s response and improving measurement precision. Figure S14D presents the pressure distribution in a two-dimensional format.

Note that, in real-world scenarios, forces are applied over a surface rather than at a single point, which reflects the conditions during the data acquisition process. Therefore, we first discretized this surface contact area with a 5-mm radius into 80-point contacts (fig. S15A). The overall pressure distribution was then calculated on the basis of the previously established EHS model for a single-point contact. To mitigate potential errors introduced by discretization, an FEM of the elastomer was developed for rectification (fig. S15B). The discrepancy between the EHS model and the FEM (fig. S15C) was corrected using a pressure-dependent correction factor, β. Figure S15D provides examples of the rectified EHS model and the corresponding β values. A second-order function was used to fit the relationship between β and external pressure (fig. S16), enabling the EHS model to be corrected under varying pressure conditions (detailed in note S3). To be noted, the FEM was only used in the early process of rectification for fitting β and excluded during generating ground-truth pressure maps, avoiding time-consuming simulations. Last, the rectified EHS model received the coordinates and force magnitude and generated a pressure map by calculating the pressure value at each position. This map reflected the pressure distribution in the physical world and was used as the ground truth for training the SATS model. Notably, the rectified EHS model can calculate pressure values spatially continuously, allowing the constructed ground-truth pressure map to have any size or shape. Therefore, to align with the pressure map inferred by the SATS model, the ground-truth pressure map was constructed to correspond with each position in the inferred map. The shape of each ground-truth pressure map was sized at 54 mm by 50 mm.

**Fig. 4.**
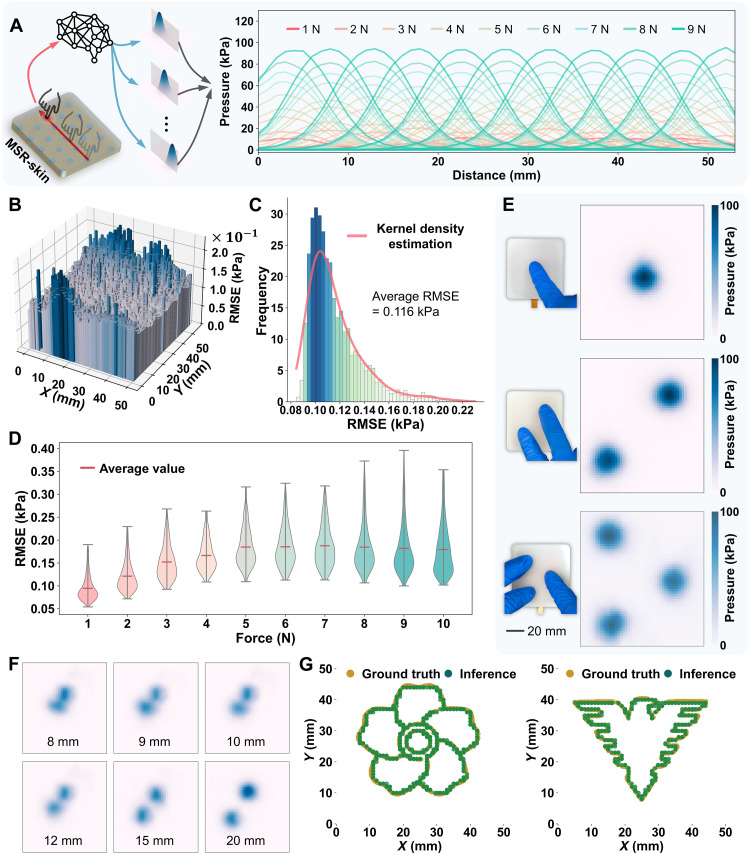
Calibration of the MSR-skin. (**A**) Section views of the inferred pressure maps under various forces applied along the symmetric line of the sensing surface. (**B**) Error distribution with respect to the pressing position. Each bar at a given position represents the RMSE averaged across the sensing surface when that position is under pressure. (**C**) Statistical analysis of the error distribution in (B). (**D**) RMSE with respect to external force. (**E**) The SATS model’s inference on pressure distributions under pressure applied at one, two, and three points, respectively. (**F**) Results from the two-point discrimination experiments. (**G**) Illustration of the MSR-skin’s SR capability in two contour-following applications, where it accurately detects contact positions along both contours.

The same dataset, collected through the robot arm and used to characterize the sensor array, was further used to train the SATS model along with the ground truths generated by the EHS model. The gradient descent method was used for training. The training process served as a calibration of the MSR-skin system. Figure S17 exhibits examples of the model’s inferences posttraining. The SATS model successfully estimated the pressure distribution across the entire sensing surface under varying forces and positions. It substantially enhanced the sensor array’s intrinsic spatial resolution by generating 54 by 50 = 2700 virtual taxels from 23 physical taxels, achieving a scale factor of 2700/23 ≈ 117. To comprehensively demonstrate the MSR-skin’s response, points along the symmetry line were sequentially pressed with a step size of 5 mm, applying forces ranging from 1 to 9 N. Responses from the five sensing units along this line were extracted and visualized (Fig. 4A). Sectional views of the inferred pressure maps along their symmetry plane are presented for clarity. By integrating responses from multiple sensing units, the SATS model effectively decouples the position and magnitude of the external force, thereby accurately estimating the pressure distribution.

To quantitatively examine the performance of this sensing system, the inference error at each position on the sensing surface was calculated. Specifically, the RMSE was computed across the entire sensing surface for a given position under varying forces. Since each position was pressed with different forces, the errors for a specific position (*x* and *y*) were averaged over the force range. This calculation process is illustrated in fig. S18 and can be formulated as

$$\text{RMSE}=\sqrt{\frac{1}{N}\sum_{i}^{N}{\left(P_{i}^{\text{gt}}-P_{i}^{\text{pred}}\right)}^{2}}$$ (2)

where *N* denotes the number of data samples collected at (*x* and *y*), and $P_{i}^{\text{gt}}$ and $P_{i}^{\text{pred}}$ are the ground-truth pressure and the model-inferred pressure at the *i*th press, respectively.

Figure 4B illustrates the calculated error distribution, revealing that the maximal error is ~0.2 kPa, and the average error is 0.116 kPa. Further statistical analysis of this error map shows that 90% of the errors are below 0.15 kPa (Fig. 4C). Comparison of interpolation methods and the SATS model, as well as the results of ablation experiments, is detailed in note S5, table S2, and fig. S19. In addition to the pressing position, the RMSE error with respect to external force was also examined. Figure 4D shows an increasing trend in error as the force rises from 1 to 10 N, with saturation occurring when the force exceeds 5 N. Furthermore, the localization error was assessed, reflecting the discrepancy between the actual pressing position and the model-inferred position. Since the SATS model does not provide the pressing position directly but rather a pressure map, the location of the maximum pressure value was identified as the pressing position. Figure S20 displays the distribution of localization error (RMSE) in relation to pressing position and external force.

Unlike the pressure map inference error, the localization error shows a descending trend as the external force increases from 1 to 10 N. This discrepancy arises from the trade-off between pressure map distortion and the signal-to-noise ratio of taxel responses. On the one hand, as the applied force increases, the corresponding pressure map exhibits greater distortion, indicating that the difference between the maximum and minimum values becomes more pronounced. This increases the difficulty of resolving fine pressure variations by the SATS model, leading to higher errors in the pressure map inference. On the other hand, force magnitude was not considered when evaluating localization errors, thereby eliminating errors introduced by force estimation. Moreover, a larger applied force enhances taxel responses, increasing the signal-to-noise ratio and improving the accuracy of pressing position localization. The average localization error across all pressing positions and force ranges was 0.73 mm, slightly less than the step size for data collection. Therefore, it can be reasonably assumed that the localization error of the sensing system is strongly related to the step size in the data collection process. It is promising to further enhance the SATS model’s localization precision and reduce the localization error. This can be achieved by using finer grid segmentation (i.e., smaller step sizes) to collect data for training an SATS model with an increased latent dimension and larger inferred map size.

A notable advantage of the proposed SATS model is the zero-shot knowledge transfer from single-point contacts to multi-point contacts. Figure 4E and movie S1 illustrate the sensing system’s inferences on pressure distributions under forces applied at one, two, and three points, respectively. Additional examples of the sensing system detecting multi-point contacts are presented in fig. S21, including the simultaneous detection of four, five, and six contact points, respectively. This capability substantially reduces the workload associated with data collection for different contact scenarios. In addition, an approach was proposed to identify the precise coordinates of contact points based on the inferred pressure maps. Specifically, the nonmaximum suppression algorithm was used to locate local maxima, which were then recognized as contact positions. Details can be found in note S6.

To explore the capability of discriminating multiple contacts, an experiment on two-point discrimination, a crucial criterion of tactile sensation (*53*), was conducted. Two points on the sensing surface were pressed while controlling the external force and interval (fig. S22A). The pressure maps inferred by the SATS model were processed using the K-means algorithm (note S6) to identify two local maxima as the predicted contact positions. The results are shown in Fig. 4F and fig. S22B. The minimal interval the SATS model could discriminate was 8 mm (approximately the interval between adjacent sensing units) under an external force greater than 25 N. It is observed that a larger external force improves distinguishability, which is consistent with results reported for a single-point contact. By this experiment, some limitations are observed as well. The SATS model faced challenges to accurately distinguishing two closely located contacts. The reasons include three points. From the physical perspective, the pressure fields of two contacts overlap with each other, leading to hard decomposition directly at the physical level. From the SATS model’s perspective, adjacent local maps are semicoupled due to the working mechanism of the self-attention module, indicating that pressure variations in one map may influence the inferred pressure distribution in a neighboring one. From the signal overlap perspective, a single-point contact typically activates three taxels, implying that two close contacts may activate the same taxels, which can compromise the accuracy of multi-point contact inference.

### Demonstration of SR tactile perception

The developed tactile sensor, with its spatial SR capability, is adept at detecting subtle patterns. Figure 4G showcases the MSR-skin’s performance in two contour-following applications. Initially, the robot arm was programmed to press each position along the contour lines of the patterns, simultaneously recording the responses of the sensor array and the corresponding coordinates. Subsequently, the time-series data from the sensor array were input into the SATS model for inference. The location of the maximal value in a pressure map was taken as the inferred pressing position, achieving an average RMSE of 0.32 mm. Notably, this position error is lower than the previously reported average RMSE of 0.73 mm in the validation part. This reduction is attributed to an averaging strategy, which computes the mean coordinates of the SATS model’s inferences at each pressing position, synthesizing temporal information along the force dimension.

**Fig. 5.**
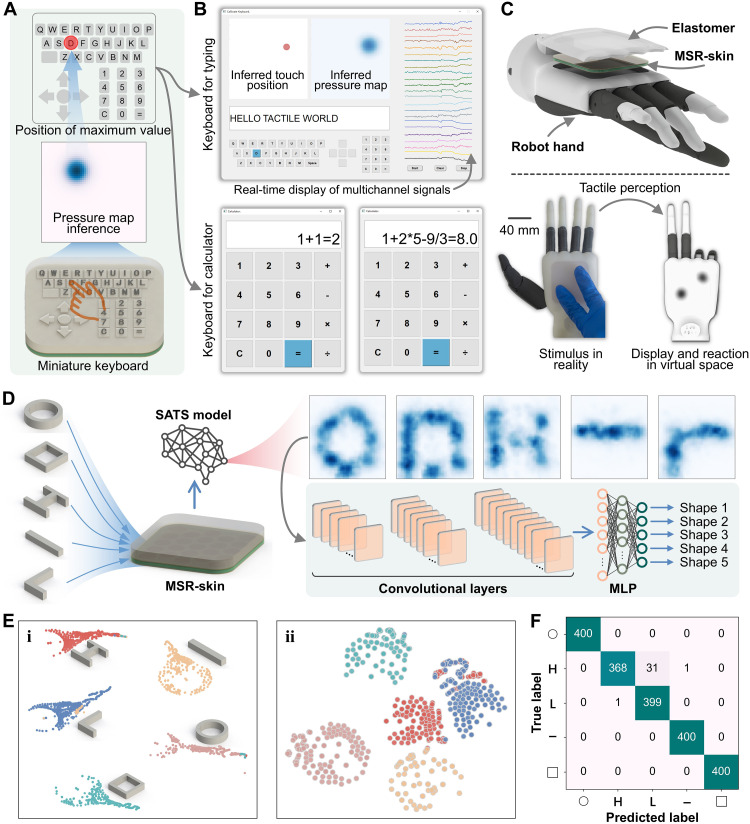
Human-machine interaction applications enhanced by SR sensation. (**A**) Miniaturization of a computer keyboard onto the sensing surface. (**B**) Realization of typing and calculator applications using the keyboard. (**C**) Integration of the tactile sensor into a robot hand, providing enhanced tactile perception and interactive applications. (**D**) Imaging and CNN-based classification of various shapes. (**E**) Dimension reduction of (i) training data and (ii) test data using the t-distributed stochastic neighbor embedding algorithm. (**F**) Results of the confusion matrix for classifying various shapes.

Besides, it is well-suited for applications requiring high integration and customization, achieving human-machine interaction tasks that require accurate localization. For instance, a miniature keyboard was customized onto a compact area of the sensing surface (Fig. 5A). When a tiny key was pressed, the SATS model accurately estimated the coordinates of the pressing position. The specific key being pressed was determined by referencing a predefined coordinate dictionary. This integrated keyboard can function as an input device for human-machine interaction, assisting a robot in understanding user intentions. Figure 5B and movie S2 illustrate the tactile sensing system’s application as a keyboard. Building on this, a calculator application was designed, as shown in movie S3.

This tactile sensor could be integrated into robots to facilitate interaction between humans and robots to provide embodied tactile perception. Figure 5C shows the tactile sensor integrated into a robot hand. With this tactile sensor, the robot hand can detect external stimuli and respond according to the type of contact (movie S4). This establishes the foundation for human-machine interaction applications. For example, using the SR capability supported by the SATS model, this tactile sensor was used to image contact shapes (Fig. 5D). In comparison, the original responses from the sensor array, shown in fig. S23, offered limited information about the pressure distribution. It is worth noting that this application to surface contact detection differs substantially from the single-point contact scenario, from which the data used to train the SATS model were collected. This created a challenge on zero-shot knowledge transfer from point contact to surface contact, which proved difficult and might have led to an imperfect performance of the SATS model. Nevertheless, it remains a substantial investigation aiming at bridging the gap between distinct applications through an efficient approach.

Subsequently, these images were accurately recognized by a CNN-based classifier, achieving an accuracy of 99.5% on the training data and 98.35% on the test data. The CNN-based classifier was designed to have a compact architecture to minimize computational complexity, ensuring resource-efficient deployment and low-latency inference (~1 ms per sample) in real-time embedded systems. Real-time recognition of different contact shapes was successfully implemented, as evidenced in movie S5. In addition, the t-distributed stochastic neighbor embedding algorithm was used to reduce the dimensionality of the classifier’s output space (Fig. 5E). Both the training and test data were distinguishable, with data of the same class clustered together and different classes separated. The classification performance of the test data, as shown in the confusion matrix, is depicted in Fig. 5F. Most shapes were correctly identified, with misclassification primarily occurring between the “H” shape and the “L” shape. A credible explanation is that these shapes share structural similarities, such as H being composed of four L shapes. In addition to large-scale shapes, the recognition of small-scale shapes was investigated, as detailed in note S7 and fig. S24. A classification accuracy of 92.87% was achieved on the test set, which is lower than that for large-scale shapes, indicating a more challenging task.

### Framework generality

The proposed computational framework for tactile SR can be deployed into several conditions, including pressure map inference and conventional coordinate and force inference. Note S8 discussed the SATS model’s effectiveness and performance in the above conditions, leveraging simulation data (figs. S25 to S30). Besides, investigating the SATS model’s interpretability further proved its powerful capability of learning both temporal and spatial characteristics (fig. S29).

The tactile sensor prototype used in this study is piezoresistive for pressure detection, with an elastomer used as the transmission medium to expand the receptive field. In the above investigation, the elastomer thickness was empirically set to 5 mm, demonstrating proper performance in subsequent tactile SR applications. The receptive field size of a sensing unit could be influenced by its intrinsic characteristics, signal-to-noise ratio, and elastomer thickness. Given an integrated sensing system where sensitivity and signal-to-noise ratio are predefined, the receptive field size can be adjusted by modifying the thickness of the elastic covering to achieve an optimal configuration. A detailed discussion of this aspect is provided in note S9 and fig. S31.

Furthermore, the proposed computational haptic paradigm can also be applied to sensors with different mechanisms and transmission mediums. For instance, TENGs in single-electrode mode can use the electric field as the transmission medium. By combining signals from adjacent taxels generated by variations in the electric field, the SATS model can accurately infer contact positions, surpassing the sensor’s physical resolution. We explored this potential by designing a TENG-based tactile sensor following the proposed computational paradigm (fig. S32 and note S10). The coordinate inference version of the SATS model was used to calibrate the fabricated sensor, and a localization error of ~1.3 mm (RMSE) was obtained, achieving an SR scale factor of approximately 120. In addition, the sensor’s high sensitivity to dynamic stimuli, a characteristic of TENGs, enabled the detection of rapid events, such as a bouncing ping-pong ball, as shown in movie S6. The SATS model further enhanced localization accuracy in these dynamic scenarios.

## DISCUSSION

### SR capability

This work investigated the possibility of computational tactile sensation and developed a framework based on deep learning techniques, enabling tactile sensors to detect SR pressure distribution. Particular attention was given to optimizing the utilization of existing sensing resources. The SATS model was designed to receive multichannel signals and estimate pressure distribution. This framework enhanced tactile sensors’ spatial resolution, surpassing physical limits. It substantially compensated for hardware limitations, simplified the structure of tactile sensors, and improved reliability and durability. In addition, the improved resolution enabled various human-machine interaction applications and contributed to the development of embodied perception. Furthermore, the universality of the proposed framework allows its application across various tactile sensor designs. Although larger errors were observed at the edges of the sensing surface due to incomplete coverage by receptive fields, it could be addressed by extending new taxels. Besides, achieving higher resolution is promising through fabricating smaller and denser taxels using advanced manufacturing techniques, enhancing the hardware’s ability to capture fine details of external stimuli. Subsequently, SR tactile sensing could be further improved by collecting higher-fidelity data and training an SATS model with modified latent feature dimensions.

### Training efficiency and scalability

The SATS model allows zero-shot knowledge transfer from single-point to multi-point contact scenarios. Training only requires single-point contact data to achieve SR sensing, and the same model can handle multi-point sensing without needing retraining. This feature increases the model’s adaptability, making it suitable for various applications. It should be noted that when two or more points are close together, the activated taxels may be the same, as if only one point were present. Thus, applying pressure to multiple points simultaneously activates the same taxels as a single-point contact, aggravating the domain shift issue between single-point and multi-point touch. In other words, when a multi-point contact involves densely distributed stimuli, the sensing performance may be compromised. This issue may be aggravated in complex contact scenarios, such as imaging different shapes as shown in Fig. 5D, which presents a knowledge transfer challenge from point contact to surface contact. Although imaging may be imperfect in pressure inference due to this challenge, improvements could be made from both software and hardware perspectives. On the one hand, additional data could be collected to fine-tune the SATS model for multi-point and surface contact detection. On the other hand, using smaller and denser taxels could enhance the sensor array’s physical resolution, thereby providing higher-quality raw data. In addition, the system is easily scalable—only a new LSTM layer is needed to integrate a new sensing unit that is added following the proposed layout, while the self-attention, local map construction, and CNN layers remain unchanged.

## METHODS

### Fabrication of the porous CNT/PDMS sponge

The porous CNT/PDMS sponge was fabricated through a standard acid etching process, using Ni foams as the porous template. Initially, a mixture of CNT (XFM19, XFNANO, China) and chloroform (10006862, Sinopharm Chemical Reagent Co. Ltd., China) was prepared at a weight ratio of 1:20. This mixture was then sonicated using an ultrasonic cell disruption system (LC-JY96-IIN, LICHEN, China) at a power of 90 W for 15 min. Subsequently, a mixture of PDMS precursor (Sylgard 184, Dow Corning, USA) and curing agent (10:1 ratio) was diluted by adding chloroform at a weight ratio of 1:3. The diluted PDMS solution was added to the CNT/chloroform mixture and stirred for about 10 min at a speed of 650 rpm at room temperature. The resulting mixture was heated and stirred (650 rpm) at 80°C to evaporate ~60% of the chloroform in a fume hood. Next, Ni foams (KSH-1011, Willtek Photoelectric Materials Co. Ltd., China) of 2-mm thicknesses were cut into circular shapes with a diameter of 10 mm using an infrared laser system (wavelength: 10.6 μm; VLS 2.50, UNIVERSAL Laser System Inc.). The Ni foams were then immersed in the CNT/chloroform/PDMS mixture for 30 s and shaken on a sieve to remove uneven or cohesive parts. After baking at 90°C for 30 min, the cured samples were immersed in a 3 M HCl solution for 20 hours to etch the Ni foam. Last, the porous CNT/PDMS sponges were obtained by washing the etched samples several times with distilled water.

### Fabrication of the sensor array

The sensor array was fabricated layer by layer. The electrode layers of the sensor array were custom-made flexible printed circuit boards fabricated by JLC Technology Group Co. Ltd., China. Then, a PET layer of 0.12-mm thickness from Dongguan Hongshun Adhesive Products Co. Ltd., China was attached to the bottom electrode layer and laser-cut by a machine (FD-300, Fengying Computer Technology Development Co. Ltd., China) to expose the electrodes. This processed PET was a mask for the blade coating of silver paste (GC-SP360-A, Shandong Jiahui Material Technology Co. Ltd., China). The prepared sensing units were directly attached to the silver paste on the bottom electrodes. A spacer was placed on the bottom layer’s edge and made from the same material as the sensing units to ensure uniform mechanical properties. The top electrode layer was prepared using the same process as the bottom layer and placed on top of the sensing units. To solidify the silver paste, this integrated sensor array was heated using a heating stage (X1010T, Xinhaomai Electronic Technology Co. Ltd., Shenzhen, China). Afterward, an elastomer made of Dragon Skin 10 (Smooth-On, USA), with a thickness of 5 mm, was placed on top of the sensor array, serving as both a protective cover and a force transmission medium.

### Characterization

An SEM (Gemini 300, Zeiss, Germany) was used to examine the surface morphology of the porous CNT/PDMS sponge. The mechanical properties of the sensing material were tested using a universal testing machine (34SC-05, Instron, USA). A mobile platform (LY100-C, Jevid Hydraulic and Pneumatic Machinery Factory, China) was used in the measurement of the receptive field. During the characterization process, an ATmega328P microcontroller (Microchip Technology Inc., USA) was used as the processor for the data acquisition module. Resistance was measured through a voltage divider at a sampling rate of 500 Hz.

### Signal acquisition of sensor array

For the 23-node tactile sensor prototype, a customized circuit board was designed and manufactured. Using the four-wire resistance measurement method, the STM32F microcontroller from STMicroelectronics (Switzerland) was used for resistance measurement and data acquisition. Each sensing unit was connected to a top electrode for current input and a bottom electrode for current output, resulting in 46 channels (23 × 2). Consequently, two multiplexers (CD74HC4067, Texas Instruments, USA) were used to control 23 channels. The schematic circuit diagram is shown in fig. S6. The sampling rate for data acquisition was set at 10 Hz.

### Data acquisition for DNN model training

To collect data for calibrating the sensor array and training the SATS model, a robot arm (UR5, Universal Robots, Denmark) equipped with a six-dimensional force sensor (Gamma IP60, ATI Industrial Automation, USA) was programmed to press each position on the sensing surface. With force feedback, the robot arm was controlled at each position to gradually increase the displacement along the normal direction of the sensing surface until a force of 10 N was reached. The robot arm was then released and moved to the next position. During this process, the coordinates of the robot arm’s end effector and the force sensor’s measured force were recorded using the rosbag tool. In addition, the responses from the sensor array were recorded, resulting in 23-channel time-series data. These two data types were aligned along the time axis using recorded timestamps and segmented using a sliding window of size 10 sample points (aligned with the LSTM’s inputs). Data from the robot arm generated ground truths, while data from the sensor array served as inputs to the SATS model.

Data were collected in the large-scale shape recognition application by randomly placing three-dimensional printed models of various shapes on the sensor array and applying random normal force on these models. This process was completed by volunteers rather than a standard compression testing machine to ensure the diversity of data distribution. Approximately 4000 samples were collected for each shape, with 90% used for training and 10% for validation. The sensor array’s responses were recorded and processed by the SATS model to infer pressure maps, which were then used as inputs to the CNN-based shape classifier.

### The DNN models

In the SATS model, a one-layer LSTM with a hidden size of 125 was used to encode the time-series signals with a window size of 10. Each sensing unit was assigned a unique LSTM to accommodate its unique characteristics. The self-attention module was implemented on the basis of the graph attention model (GAT) (*54*), with two GAT layers, each having a hidden size of 125. Before input into the self-attention module, features from the LSTMs of all sensing units were structured as a graph, of which the vertices were encoded features, and an adjacent matrix defined the edges. For each sensing unit, features from the LSTM model and the self-attention module were concatenated and input into the local map construction module, a three-layer MLP (250 by 375 by 500 by 195). Each layer of the MLP was followed by a LeakyReLU (*55*) activation function. The output from the MLP was resized into a two-dimensional matrix of size 13 by 15 to form the local pressure map. These local maps were then masked and merged according to their positions (the locations of the corresponding sensing units) to construct the overall pressure map. Last, the overall map was processed by a two-layer CNN to obtain the final inference of the pressure distribution, with each layer of the CNN using a convolutional kernel of size 3 by 3 and the first layer followed by a LeakyReLU activation function. The constructed overall pressure map is formulated as a matrix of shape 54 by 50, of which each value is regarded as a virtual taxel, contributing to 2700 virtual taxels in total. The SATS model was trained using the adaptive moment estimation (Adam) optimizer with a learning rate of 0.0064 and a batch size of 2048 for 200 epochs. The mean square error was used as the loss function.

In shape recognition, since the outputs from the SATS model were two-dimensional matrices representing the pressure distributions induced by different shapes, a CNN-based classifier was developed to recognize these shapes. This classifier included three convolutional layers, with output channels of 16, 32, and 64, respectively, and a kernel size of 3 by 3. A max pooling layer with a kernel size of 2 by 2 followed each convolutional layer. The outputs from the last max pooling layer were flattened and fed into an MLP (256 by 64 by 5) for classification. All convolutional layers and each layer of the MLP, except for the final layer, were followed by the LeakyReLU activation function. The model was trained using the Adam optimizer with a learning rate of 0.001. Cross-entropy loss was used as the loss function.

The models mentioned above were implemented in Python 3.11.5 using the PyTorch framework (version 2.1.1). Training was conducted on an Nvidia GeForce RTX 4090 graphics processing unit under Ubuntu 20.04. The time cost of training the SATS model for one epoch was about 16 s, leading to about 53 min for 200 epochs.

### Finite element analysis

Since the elastomer was a hyperelastic material, the Mooney-Rivlin model was used to simulate its properties. The density was set as 1 × 10^−3^ g/cm^3^. Parameters in the Mooney-Rivlin model were set as follows: *C*_10_ = 0.144, *C*_10_ = 0.036, and *D*_1_ = 0. In the simulation, a cylindrical body with a diameter of 100 mm and a thickness of 5 mm was created. A uniform load was applied over a circular region with a diameter of 10 mm at the center of its upper surface. Pressure distribution on the bottom was then recorded.

## Supplementary Materials

### The PDF file includes:

Notes S1 to S10 Figs. S1 to S32 Tables S1 and S2 Legends for movies S1 to S6 References
