# Supplementary material for: Super-resolution tactile sensor arrays with sparse units enabled by deep learning
Source: Sci Adv. 2025 Jul 2;11(27):eadv2124. doi: 10.1126/sciadv.adv2124 (PMC12219507; doi:10.1126/sciadv.adv2124)
Supplement: Supplementary file 1 — Notes S1 to S10 Figs. S1 to S32 Tables S1 and S2 Legends for movies S1 to S6 References [file sciadv.adv2124_sm.pdf]

Science Advances
AAAS

Supplementary Materials for

Super-resolution tactile sensor arrays with sparse units enabled by deep learning

Depeng Kong *et al.*

Corresponding author: Kaichen Xu, xukc@zju.edu.cn; Geng Yang, yanggeng@zju.edu.cn

Sci. Adv. **11**, eadv2124 (2025)
DOI: 10.1126/sciadv.adv2124

The PDF file includes:

Notes S1 to S10
Figs. S1 to S32
Tables S1 and S2
Legends for movies S1 to S6
References

Other Supplementary Material for this manuscript includes the following:

Movies S1 to S6

Supplementary Text

Note S1. Calculation of the SR scale factor

By utilizing a super-resolution algorithm, areas between physical taxels can also detect external stimuli by generating virtual taxels. The super-resolution scale factor is defined as the ratio of virtual taxels to physical taxels. For methods that directly report the density (number) of generated virtual taxels, the scale factor is calculated by

$$\alpha = \frac{N_v}{N_r}, \tag{S1}$$

where N_r and N_v indicate the number of real taxels and virtual taxels, respectively. For methods that did not provide the number of virtual taxels but instead reported localization error, the scale factor is calculated as

$$\alpha = \frac{N_v}{N_r} = \frac{S}{N_r \pi \epsilon^2}, \tag{S2}$$

where S represents the sensing area of the sensor array, ϵ is the localization error in the form of root-mean-square error (RMSE).

Note S2. Layout optimization of general conditions

An optimization problem is formulated in equation (1) to search for the optimal layout of a sensor array using the PSO algorithm. However, PSO is not the only approach; other general optimization algorithms, such as Bayesian optimization (BO), genetic algorithm (GA), and simulated annealing (SA), can also be employed to solve this problem. This study compared several methods, including PSO, BO, GA, and SA. The radii of the three receptive fields were set to r=1 for simplification. Each algorithm was used as the optimizer for solving the problem defined in equation (1). The convergence curves are shown in Fig. S2A. After several iterations, all algorithms converged to the same result, where three taxels were positioned at the vertices of an equilateral triangle, with side lengths equal to the receptive field radius r, as shown in Fig. S2B. Among the methods, PSO and SA exhibited the best performance, with the fastest convergence and the largest union area. Finally, the PSO was selected as the optimization algorithm for its better performance than SA.

In the current setup, the three sensing units are considered as the basic optimization unit for the array layout, with the assumption that the radii of the three receptive fields are equal (i.e., radius ratio=1:1:1). This optimization approach can be extended to accommodate conditions where the radii of the receptive fields differ. To explore this, we investigated four different scenarios with the following radius ratios: 1.5:1:1, 1.5:1.5:1, 1.5:1.2:1, and 1.5:1.3:1.1. The PSO algorithm was employed for the optimization process. Results are shown in Fig. S3. For each condition, the optimization process converged to a specific configuration. However, we observed suboptimal utilization of some receptive fields, especially for smaller radii. To quantify this, we define the sensing resource utilization rate in the context of tactile super-resolution as follows:

$$\alpha = \frac{A_1 \cup A_2 \cup A_3}{A_1 + A_2 + A_3}, \qquad \text{(S3)}$$

This equation represents the ratio of the union area after optimization, which satisfies the requirement for overlapping receptive fields, to the total area of all receptive fields. In tactile super-resolution, the goal is to use the fewest sensing units possible to cover the largest area, which can be effectively expressed by the resource utilization rate. For the radius ratios of 1:1:1, 1.5:1:1, 1.5:1.5:1, 1.5:1.2:1, and 1.5:1.3:1.1, the calculated utilization rates are α=68.4%, α=58.7%, α=66.9%, α=62.6%, and α=63.6%, respectively. Therefore, it is recommended to maintain uniformity among the used sensing units to ensure equal receptive field sizes (radius ratio=1:1:1), thereby improving the resource utilization rate.

Note S3. Formulation of the self-attention module

The self-attention module is designed to aggregate information from adjacent taxels to the current taxel. By combining multi-source information from non-collinear units, spatial information (e.g., pressing position) can be inferred. After the LSTM module encodes the data, the local feature, denoted as h_i, of a given sensing unit *i* is acquired. The set of the adjacent units of unit *i* is denoted as $\mathcal{N}_i$. First, an attention coefficient between unit *i* and each of its adjacent units in $\mathcal{N}_i$ is calculated by

$$e_{ij} = a\big([Wh_i \parallel Wh_j]\big), \forall j \in \mathcal{N}_i, \tag{S4}$$

where *W* is a matrix for linear mapping, symbol ‖ represents the concatenation operation, *a* is a function that maps the concatenated feature into a real number, e_{ij} is the calculated attention coefficient, which denotes the importance of unit *j*'s feature to unit *i*. Then, a normalization operation across all choices of *j* is applied using the *softmax* function *(56)*

$$\alpha_{ij} = \frac{\exp\left(LeakyReLU(e_{ij})\right)}{\sum_{k\in\mathcal{N}_i}\exp\left(LeakyReLU(e_{ik})\right)}, \tag{S5}$$

Once obtained, a linear combination of the features is computed using the normalized attention coefficients by

$$h'_i = \omega\big(\textstyle\sum_{j\in\mathcal{N}_i}\alpha_{ij}Wh_j\big), \tag{S6}$$

where ω is a nonlinear activation function (exponential linear unit, ELU *(57)*). h'_i is taken as the aggregation feature for unit *i*. This whole process is denoted as $f(\cdot)$, as shown in Fig. 2E.

Note S4. The elastic half-space model and its rectification for ground truth generation

The elastic half-space (EHS) describes a semi-infinite space filled with elastomer (Fig. S14A). When a point within this space is subjected to pressure, the stress at another point inside the elastomer is inversely proportional to the distance between them. According to Boussinesq's solution, the normal stress σ_{zz} at a location (*x, y, z*) within the elastomer can be expressed as *(58)*

$$\sigma_{zz} = -\frac{3Fz^3}{2\pi r^5}, \tag{S7}$$

where $r = \sqrt{x^2 + y^2 + z^2}$, and *F* represents the normal force along the Z axis.

To approximate a surface contact using the above equation for point contact, we can discretize the surface contact into *N* multiple point contacts. Then, the approximated pressure distribution $\tilde{\sigma}_{zz}$ of the surface contact can be calculated by

$$\tilde{\sigma}_{zz} = -\sum_{i=1}^{N} \frac{3F_i z^3}{2\pi r^5}, \tag{S8}$$

To compensate for the potential errors introduced by discretization, a rectification factor β is introduced as

$$\bar{\sigma}_{zz} = \tilde{\sigma}_{zz}\beta(p) = -\sum_{i=1}^{N} \frac{3F_i z^3}{2\pi r^5}\beta(p) \approx \sigma_{zz}, \tag{S9}$$

where p represents the average pressure induced by external force over the contact surface. The relationship between β and p was established with the aid of a finite element module (Fig. S16).

Note S5. Comparison results of interpolation methods and ablation experiments

The data collected for training the SATS model were randomly divided into training and test sets in a ratio of 0.85:0.15. The training set was used to train the SATS model. To comprehensively evaluate the performance of the sensing system comprising the tactile sensor and the SATS model, we tested the pre-trained SATS model's performance on the training set, test set, and their union set. Fig. 4, (B to D) and Fig. S18 present the results obtained from the union set. The average RMSE for the training, test, and union sets is 0.113 kPa, 0.13 kPa, and 0.116 kPa, respectively.

Additionally, the performance of the SATS model was compared with its variants and interpolation methods. The LSTM, self-attention module, and CNN module were removed, respectively, to obtain three variants denoted as SATS-noLSTM, SATS-noAttention, and SATS-noCNN. Furthermore, the local map construction module was replaced by an end-to-end overall map construction module that directly estimated the overall pressure map, resulting in another variant, SATS-overall. For the interpolation methods, linear, quadratic, cubic, and Gaussian interpolations were used to fit the pressure distribution based on the responses from 23 taxels. The resistance change calculated from each taxel was first converted to pressure using the fitted curve in Fig. S25E. Subsequently, the pressure distribution across the sensing surface was calculated using these interpolation methods.

The above methods were tested using the training, test, and union sets, respectively. Results are summarized in Table S2. Compared with learning methods, interpolation methods exhibited relatively poor performance as they showed larger errors. This is because interpolation methods typically rely on simple mathematical functions to approximate the response characteristics of each sensing unit, lacking a learning process that adapts to individual unit properties. Besides, these methods do not adequately account for variations among different taxels or the cooperation of adjacent taxels. This limitation is evident in the error distribution shown in Fig. 19, where all interpolation methods exhibit significant inference errors at the left edge of the sensing surface. One credible explanation is that the sensing units in that region exhibit distinct response characteristics, which makes it challenging to align them with the calibration results shown in Fig. S25E. Consequently, these interpolation methods fail to effectively utilize the original sensor responses to infer the pressure distribution. Variants of the SATS model demonstrated significantly better performance with smaller errors. Among them, the SATS-noAttention reported the worst performance, underscoring the importance of the self-attention module. Other variants also

exhibited relatively inferior performance compared to the original SATS model, illustrating each module's indispensability in the SATS model.

Note S6. Recognition of contact points in multi-point contact scenarios

The proposed sensing system directly produces pressure maps using the SATS model, providing rich and original information reflecting external contacts to help robots better perceive their environment. In the case of single-point contact, the contact position is determined by identifying the global maximum in the pressure map. For multi-point contacts, multiple local maxima need to be detected. In this work, we present two methods for this purpose: the non-maximum suppression (NMS) algorithm as a general approach, and the K-means algorithm specifically for the two-point discrimination experiment.

The NMS algorithm iteratively examines whether a given position in the pressure map represents a local maximum within a sliding window. If so, the position is recorded as a contact point; otherwise, the algorithm proceeds to the next position. By doing so, the contact positions in the pressure maps shown in Fig. S21A were accurately identified, as shown in Fig. S21B. Besides, it is observed that the average position error increases with the number of contact points, indicating that detecting multiple simultaneous contacts is more challenging. As the number of contact points grows and the distance between them decreases, interference among their pressure field deteriorates, resulting in signal overlap and, consequently, greater position errors.

The K-means algorithm was employed for two-point discrimination. First, the pressure map inferred by the SATS model is filtered by removing values less than six times the average. This procedure excludes small values and, of course, not the local maxima. The coordinates of the remaining values are then input into the K-means algorithm to obtain two clusters representing the two contact regions. For each cluster, the position corresponding to the maximum values in the pressure map is taken as the contact location. Fig. S22B presents the results calculated using this procedure.

When two contact points are too close, they might produce a single overall maximum instead of two. In such cases, the calculated contact points might be near to each other or even overlap despite the K-means algorithm identifying two clusters (Fig. S22B, lower left). The SATS model considered this a non-distinguishable condition for the two-point contact case.

Note S7. Recognition of small-scale shapes

An experiment on small-scale shape recognition was conducted to further evaluate the sensing system's capability for multi-scale tactile perception. Five additional small-scale shapes, including rhombus, triangle, square, ellipse, and pentagram, were used to apply pressure on the sensing surface. These shapes were designed to be smaller than the distance between taxels and had a circumcircle diameter of 10 mm, as shown in Fig. S24A. Six positions on the sensing surface were selected in consideration of the geometrical structure of the sensing array. Specifically, positions directly above a taxel and between three taxels were chosen to ensure diversity in the collected data. A compression testing machine was used to apply force gradually from 0 N to 10 N while data from the sensing array were simultaneously recorded.

Fig. S24B (first row) shows the pressure maps inferred by the SATS model, which are visually difficult to distinguish. After applying Laplace and Gaussian convolution kernels to convolve these maps, the differences between shapes become more discernible, as shown in Fig. S24B (second row). For simple shapes such as the rhombus, triangle, and ellipse, some geometric features, like edges and corners, can be observed. However, for more complex shapes like the pentagram, the SATS model fails to capture distinct local features such as its five horns. Unlike large-scale shapes that stimulate widely distributed taxels, small-scale shapes primarily activate nearby taxels, especially when they are smaller than the distance between taxels. This poses a huge challenge for the SATS model to accurately reconstruct their shapes. Actually, this is a challenging task for most tactile sensing systems. Besides, some local features of these small-scale shapes are even smaller than the resolution of the virtual taxels (1 mm), making precise shape reconstruction even more difficult.

4000 samples were collected for each shape, resulting in a total of 20,000 samples, of which 85% were used for training and 15% for testing randomly. These data were then processed through the pre-trained SATS model to obtain their pressure map features. Subsequently, the training data were used to train the same CNN-based classifier shown in Fig. 5D, which was then evaluated on the test data. Fig. S24C presents the dimensionality reduction results of the classifier's output space, where most data points were well-clustered according to their classes. At the same time, some were misclassified, highlighting the challenge of small-scale shape recognition. Ultimately, an accuracy of 92.87% was achieved on the test data, detailed in Fig. S24D, which is lower than that for large-scale shapes, indicating a more difficult recognition task. Nevertheless, this challenge

could be mitigated by employing a denser sensing array or collecting higher-resolution data for training the SATS model.

Note S8. Further investigation using simulated data

Inference of Pressure Map: The proposed tactile sensor, equipped with the SATS model, can directly generate pressure maps. Furthermore, the elaborately designed SATS model enables the transfer of knowledge learned from single-point touch to multi-point touch without requiring extra training. To further evaluate the performance of the SATS model, a simulation model of the tactile sensor array, based on the EHS model, was developed, as shown in Fig. S25, A and B. The sensing surface was divided into a grid (Fig. S25C), with each point on the grid serving as a position for pressing to generate simulation data. At each position, the simulated force was applied following the pattern shown in Fig. S25D. The sensor response in the simulation model was consistent with that observed in real tests. Fig. S25E illustrates the simulated sensor response curve to pressure obtained by fitting the tested response data. Noting that $y_{x=0} \approx 3.1$ simulated the static measurement error. The ground truth pressure distribution was generated directly by the EHS model (e.g., Fig. S25F). It should be noted that only one position was pressed at a time during the simulation. All positions were pressed according to the above setup for data collection (generation).

The SATS model was trained using the aforementioned simulation data. The RMSE for pressing each position was calculated as presented in Fig. S18, with an average value of 0.034 kPa, as shown in Fig. S26. Fig. S27 displays several examples of the SATS model's inferences, along with the corresponding ground truths and error distributions. It was observed that while larger pressures resulted in larger absolute errors, the relative error was maintained below 2.4% (maximum pressure to maximum pressure). Furthermore, Fig. S28 illustrates the SATS model's performance when directly applied to multi-point touch scenarios. Although relatively larger errors were observed, the SATS model remained effective under these conditions, reporting a relative error of less than 10% (maximum pressure to maximum pressure) in a three-point contact scenario. While obtaining ground truth pressure distribution under real-world conditions was challenging, the simulation results provided valuable insights.

Theoretically, the self-attention module enhances the SATS model's ability for spatial perception. To validate the effectiveness of the self-attention module, a specific sensing unit (the orange one in Fig. S29A) and its corresponding encoded features were selected for analysis. The area surrounding this sensing unit was pressed to stimulate it, and the features encoded before and after the self-attention module were recorded, respectively. The t-SNE algorithm was used to reduce the dimensionality of these features to a two-dimensional space (Fig. S29, B and C). The

features before the self-attention-based information sharing could only perceive distance and failed to distinguish the pressing positions (Fig. S29B). In contrast, the features after the self-attention module effectively decoupled spatial information. Fig. S29C shows the correspondence between data points in the two-dimensional space and pressing position, demonstrating the powerful spatial encoding capability of the self-attention module.

Inference of Coordinates and Force: Existing studies on tactile super-resolution primarily focus on localization estimation, specifically determining the coordinates of a contact point. However, a significant limitation of these approaches is that they only function effectively in single-point contact scenarios, failing when faced with multi-point contacts. This is attributed to both the design of the model structure and training data. Without an internal structure design, an end-to-end model outputs only coordinates and the force of an external stimulus in the form of a three-dimensional vector. This model can only handle single-point contacts after being trained with single-point contact data. Data of multi-point contact must be collected to train a new model whose output layer has been modified to accommodate multi-point contact scenarios. The proposed SATS model overcomes this limitation by effectively transferring knowledge learned from single-point contact to multi-point contact, leveraging the local receptive field enhanced by the self-attention mechanism. This capability is crucial for real-world applications where multi-point contact is prevalent. Additionally, the calibration data required could be significantly reduced since only single-point contact data is necessary. In practice, collecting data that encompasses all possible multi-point contact conditions is nearly impossible due to the curse of dimensionalities.

Despite the limitations of estimating coordinates for single-point contact, this approach remains relevant to tactile super-resolution and often yields an impressive scale factor. Consequently, the feasibility of applying the proposed super-resolution framework to this task was also explored. First, the SATS model was modified by replacing the local map reconstruction module with a regression module (a three-layer MLP) to infer contact position (coordinates) and force. Subsequently, 5000 positions on the sensing surface were randomly sampled for pressing, with force varying according to the paradigm in Fig. S25D. The modified SATS model was then trained. It was observed that greater force resulted in reduced position error (Fig. S30A). The spatial distribution of position errors under different forces is shown in Fig. S30B. Position accuracy improves as the force increases, with an average error of 0.12 mm (RMSE) over the whole force range. Under an external force of 8 N, this system achieved a maximal SR scale factor

of 19547, extensively surpassing the current state-of-the-art. A similar trend was observed in force inference, with an average force error of 0.035 N. With its localization capability, the system can accurately reconstruct complex and fine patterns (Fig. S30D) in contour-following applications. The results in the inference of coordinates and force further demonstrated the generality of the proposed tactile SR framework, illustrating its strong potential.

Note S9. Investigation of the optimal receptive filed size

The receptive field size of a sensing unit is influenced by its intrinsic sensing properties, the signal-to-noise ratio (SNR), and the thickness of the elastic covering. Given a predefined fabrication process, the sensing properties, such as sensitivity, remain constant. The SNR is typically affected by the magnitude of the external stimulus, while the thickness of the elastic covering can be easily adjusted to modify the receptive field size. To account for environmental noise, we assume a response threshold beyond which the signal is considered valid. For instance, a threshold of 0.15 implies that only when the relative resistance change ($\Delta R/R_0$) exceeds 15% is the response deemed effective. Since the response of the sensing unit is negatively correlated with the distance from the applied force, the boundary of the receptive field is determined by the unit's response threshold.

To explore the optimal receptive field size, we examined its dependence on the response threshold and the thickness of the elastic covering. In a specific setup where the threshold is set to 0.15, and an external force of 5 N is applied, the radius of the receptive field under different elastomer thicknesses is presented in Fig. S31A. The results indicate that the maximum radius of 11.6 mm occurs at an elastomer thickness of 5 mm. Subsequently, with the thickness fixed at 5 mm, the threshold was varied, producing the results shown in Fig. S31B. These results suggest that a lower threshold, corresponding to higher sensitivity, leads to a larger receptive field.

In summary, the threshold and thickness exhibit a coupled influence on the receptive field size, as illustrated in Fig. S31C. Additionally, given that external force influences the sensing unit's response, the magnitude of the applied force is also considered in Fig. S31C. The results demonstrate that for a given threshold (primarily determined by the sensing unit's sensitivity) and a specific force magnitude, an optimal elastomer thickness exists that maximizes the receptive field size.

Note S10. Validation of the proposed computational paradigm on a TENG-based tactile sensor

Triboelectric nanogenerators (TENGs) in single-electrode mode are well-suited for detecting dynamic stimuli and excel in sensing subtle dynamic touches. TENGs operate on the principle of electrostatic induction, allowing the electric field to act as a transmission medium, effectively enlarging the receptive field of each sensing unit. To explore the potential of the proposed computational paradigm, a 23-taxel TENG-based tactile sensor array was fabricated, with the receptive field radius of a single taxel determined to be 15 mm. This sensor was constructed as a flexible printed circuit board with a three-layer structure (Fig. S32A), providing flexibility for deployment on various curved surfaces (Fig. S32B). The Arduino development board was programmed to collect data from this sensor (Fig. S32C), outputting 23-channel signals for subsequent data processing and super-resolution.

The SATS model was adapted by replacing the local map reconstruction module with a three-layer MLP regression module to infer contact positions (coordinates). After training the modified SATS model using the Adam optimizer with a learning rate of 0.002 and a batch size of 2048, the localization error was maintained around 1.3 mm (RMSE), achieving a super-resolution scale factor of approximately 120. Leveraging the TENG's rapid dynamic response and enhanced spatial resolution, this system accurately locates external contacts, tracks contact trajectories, and even detects the position of a bouncing ping-pong ball slighter than 3 g. These capabilities are demonstrated in Video S6.

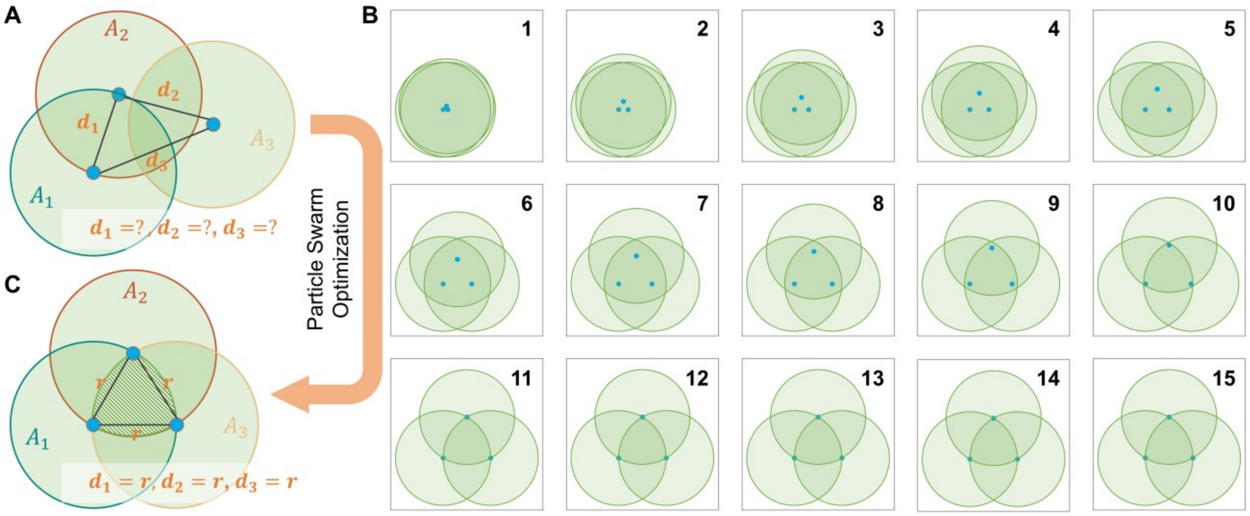

Fig. S1. Optimization of the sensing units' layout. (a) Problem statement for the optimization process. (b) Optimization process using the particle swarm optimization algorithm. (c) The optimization result, demonstrating the optimal layout is achieved when $d_1 = d_2 = d_3 = r$.

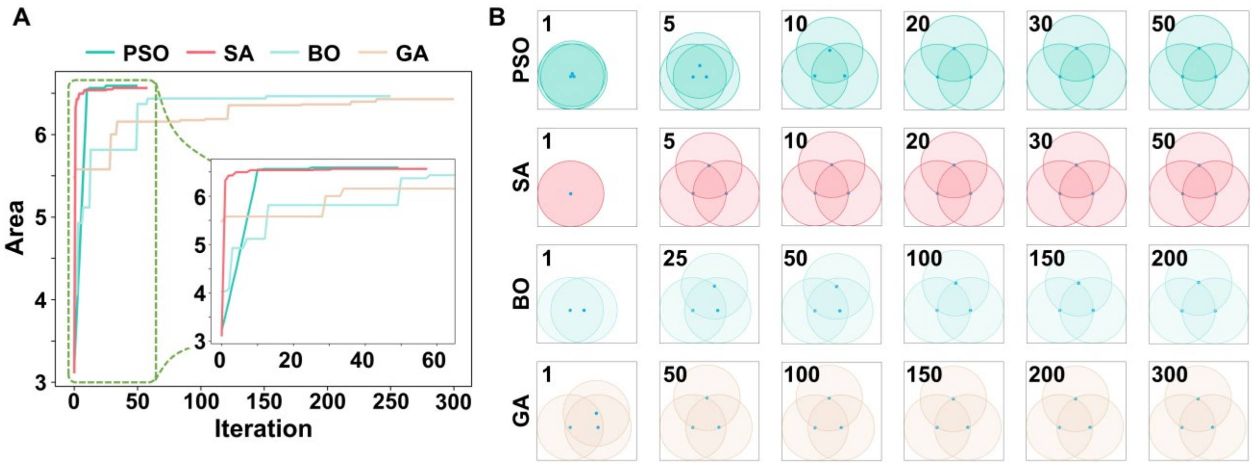

Fig. S2. Comparison of various optimization methods for the layout optimization problem. (A) Convergence curves and (B) convergence results for different optimization methods.

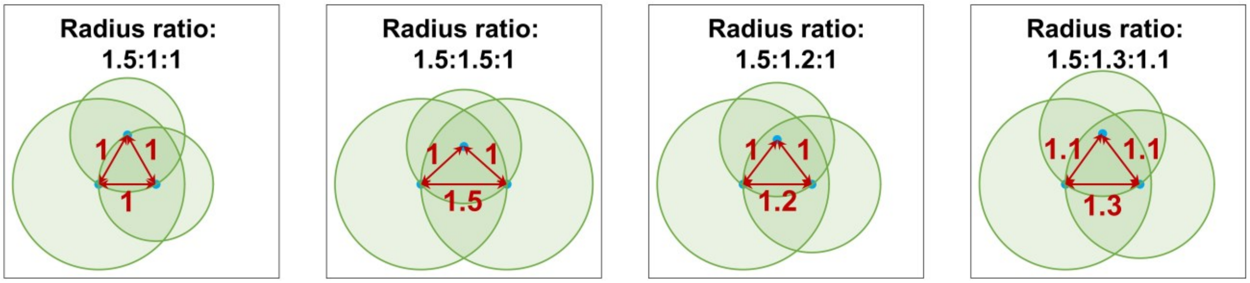

Fig. S3. The layout optimization results of different radius ratios.

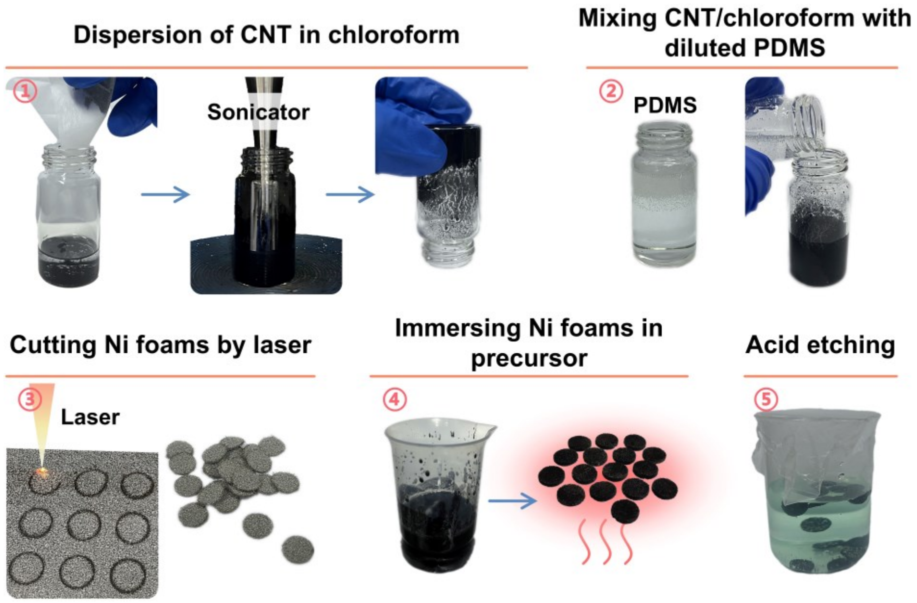

Fig. S4. Fabrication process of the CNT/PDMS sponge.

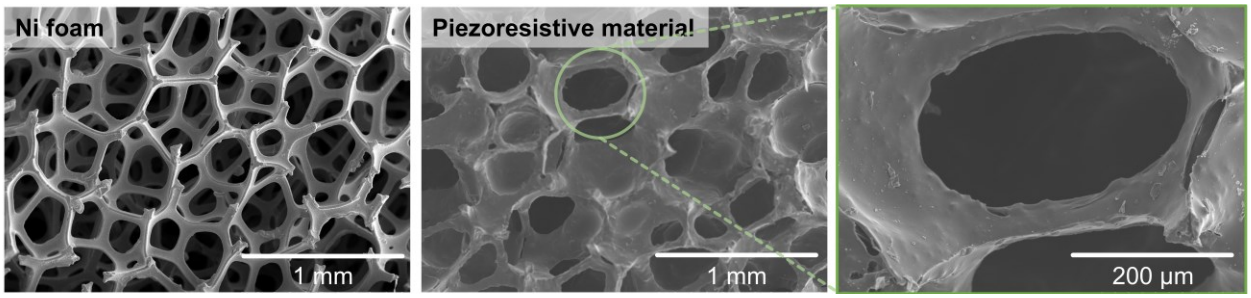

Fig. S5. SEM images of the Ni foam and fabricated porous CNT/PDMS sponge, demonstrating the porous structure.

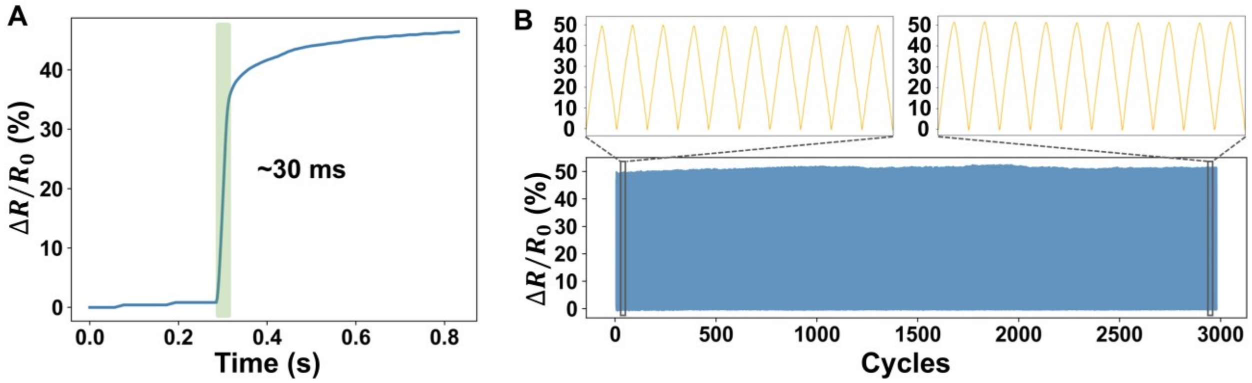

Fig. S6. Characterization of the sensing unit. (A) Response time of the sensing unit. (B) Durability test over 3000 loading/unloading cycles (amplitude ~60 kPa, and frequency ~0.3 Hz).

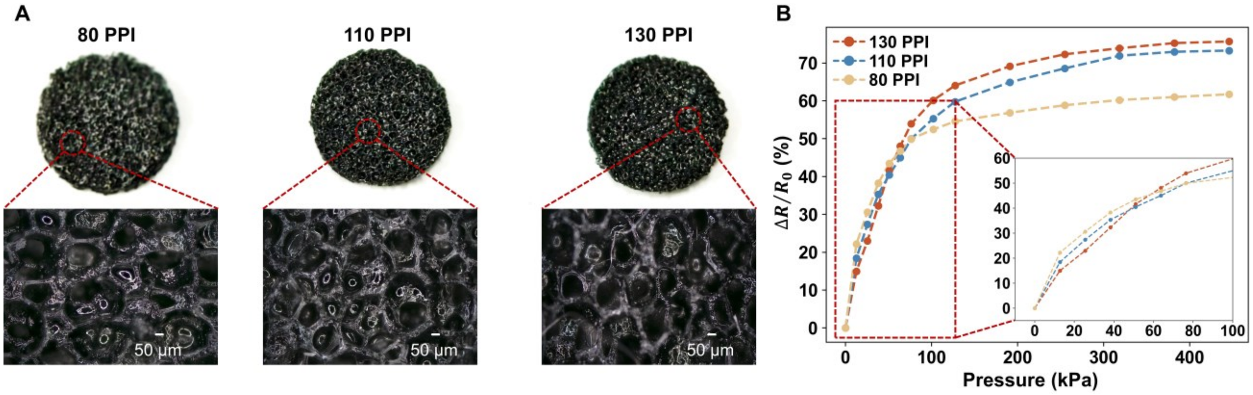

Fig. S7. Investigation of fabricating the CNT/PDMS sponges using Ni foams of various porosities. (A) Photographs of fabricated CNT/PDMS sponges with diverse porosities. (B) Relative resistance changes of these CNT/PDMS sponges under varying pressures.

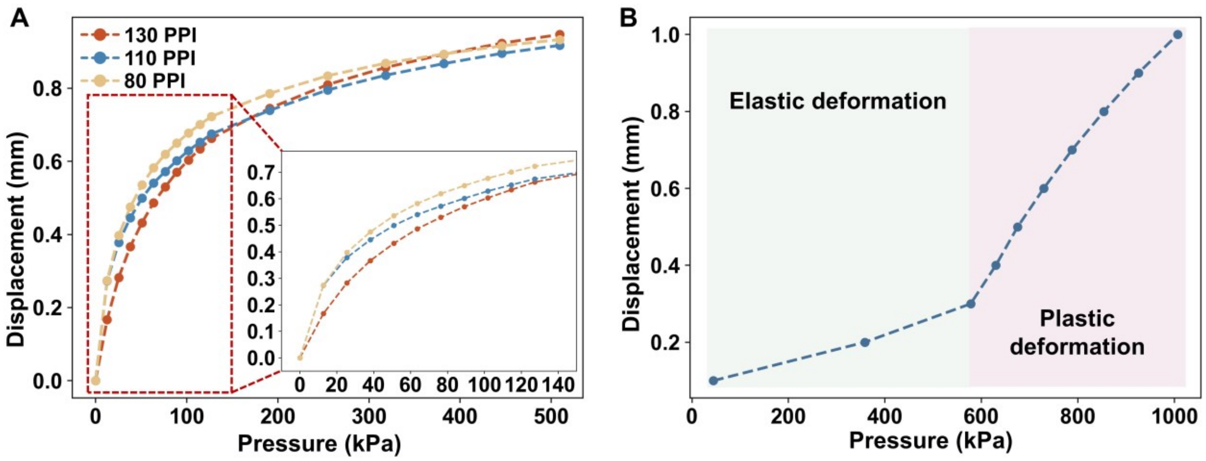

Fig. S8. Mechanical properties of (A) CNT/PDMS sponges and (B) pure Ni foam.

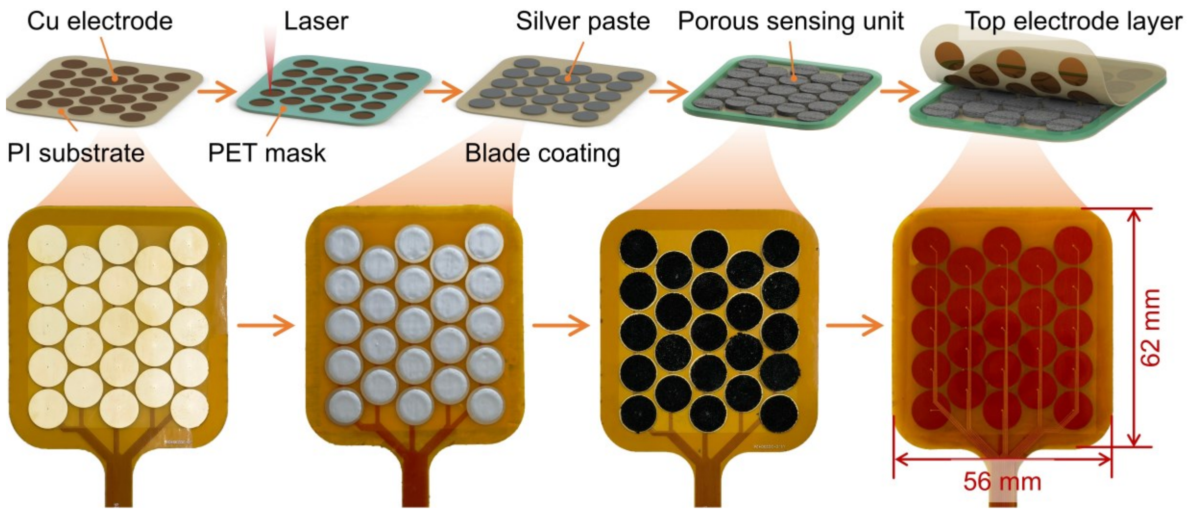

Fig. S9. Photographs of the sensor array prototype during fabrication.

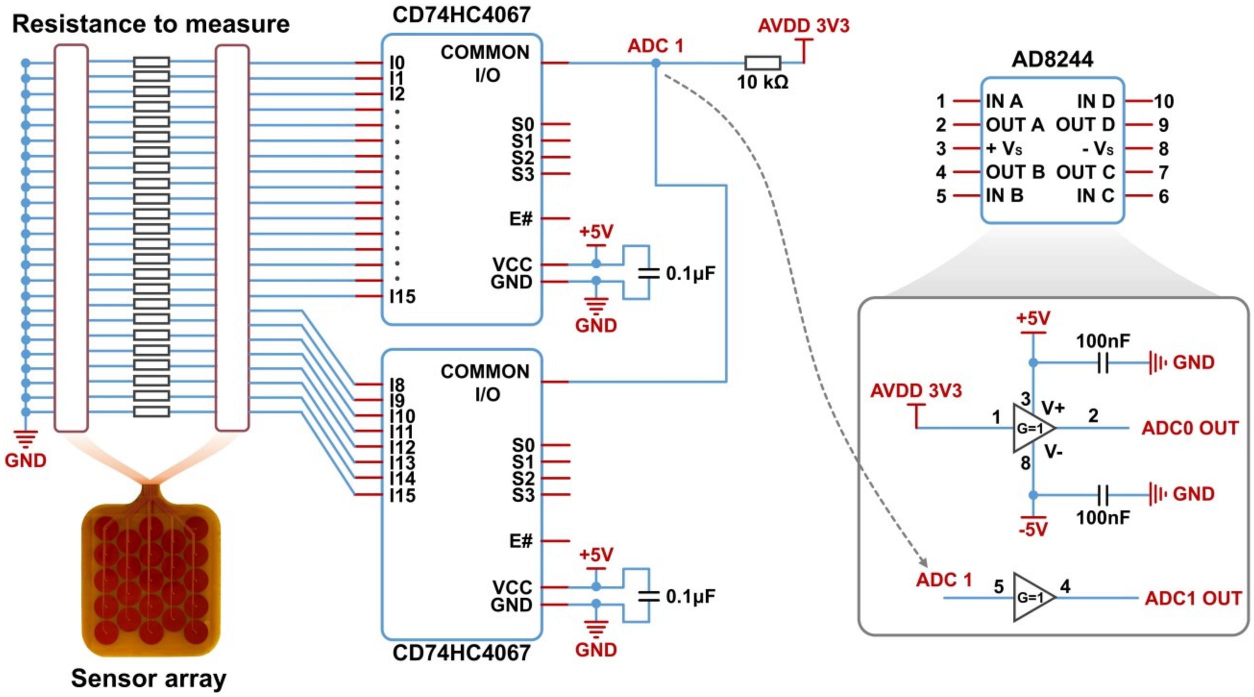

Fig. S10. Schematic circuit diagram of the resistance measurement and data collection circuit board. The S0-3 ports of the multiplexer are connected to the control port of the STM32F and are responsible for selecting specific channels for resistance measurement based on control commands. Capacitors are installed in the circuit to filter high-frequency noise and stabilize the power supply signal. In practice, the 23 channels are selected sequentially, and the resistance values are measured and recorded in real time. The data is then transmitted to the host computer via serial communication.

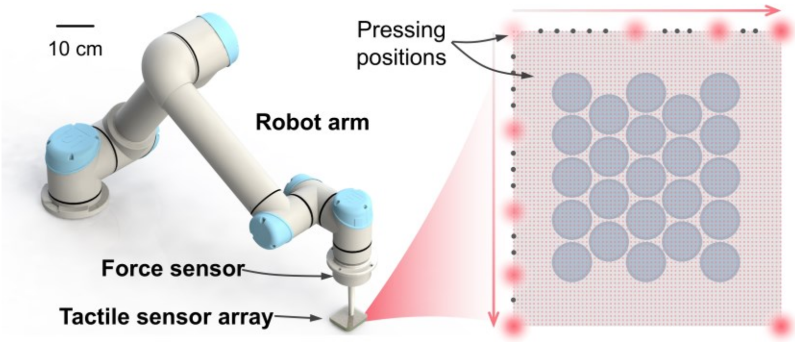

Fig. S11. Data collection using a programmed robot arm for calibrating the tactile sensor array.

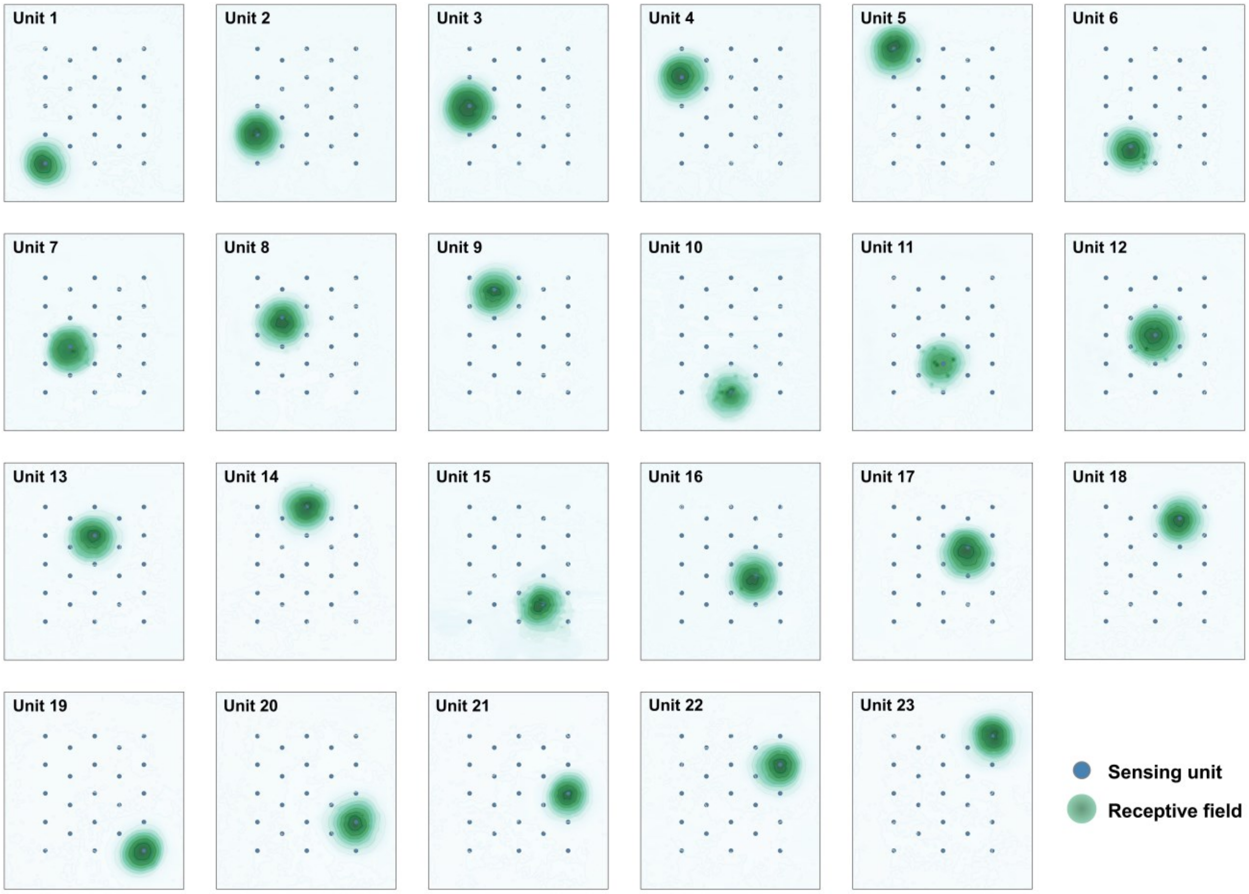

Fig. S12. Receptive fields of taxels in the sensor array.

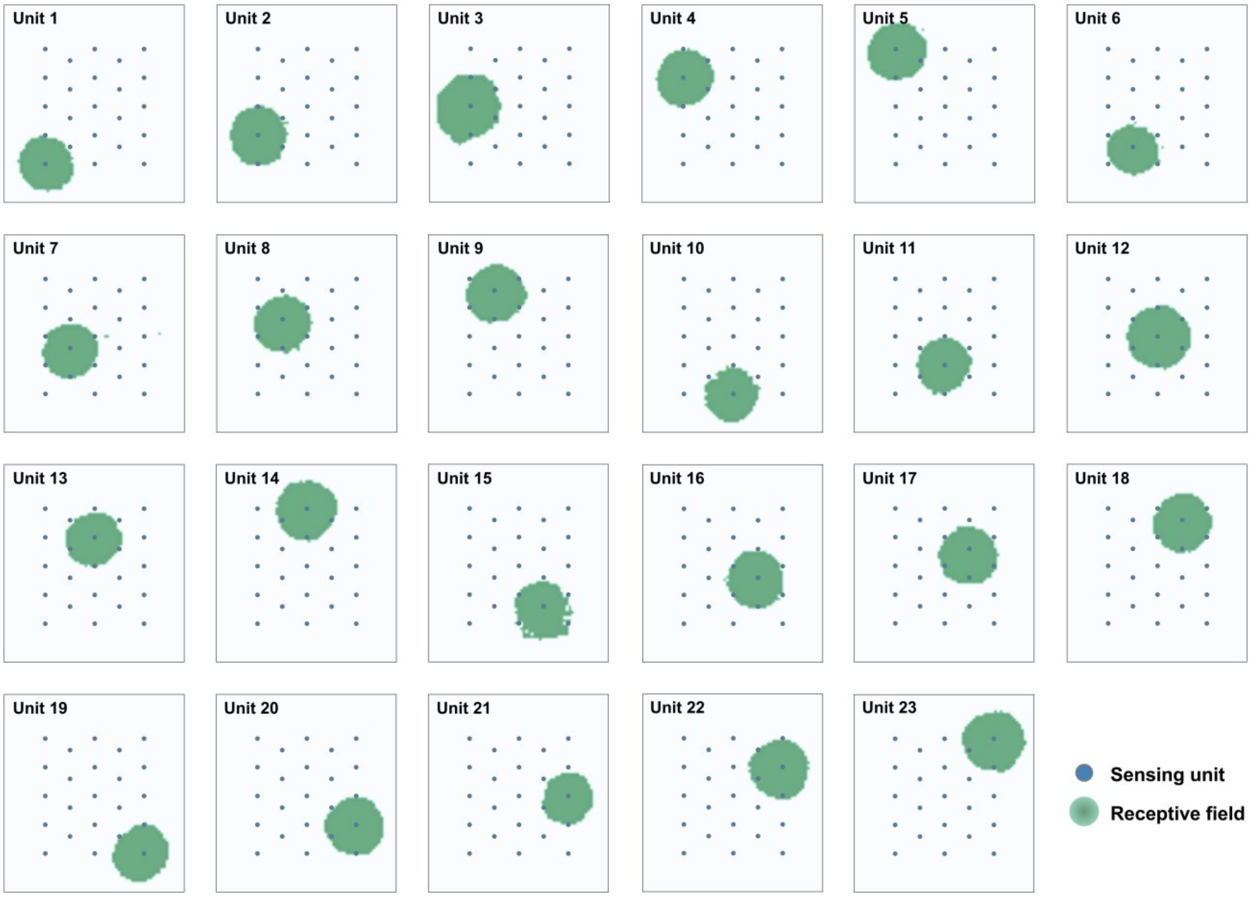

Fig. S13. Filtered receptive fields of taxels in the sensor array.

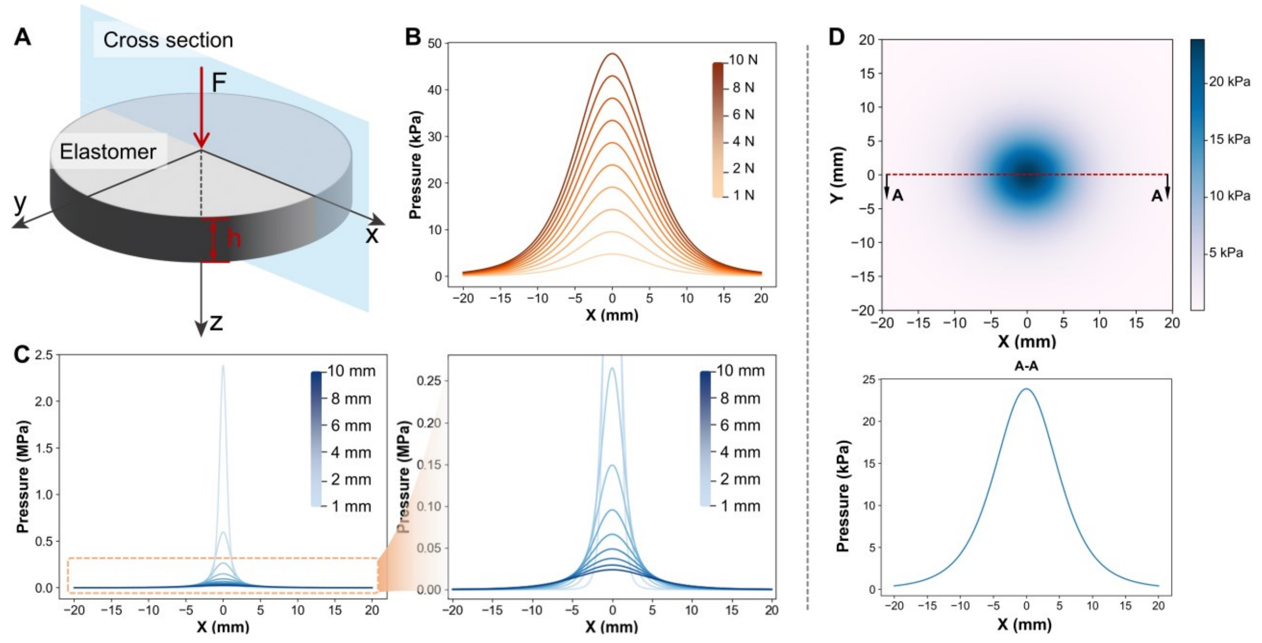

Fig. S14. Illustration of the elastic half-space. (A) Coordinate system setup showing an external force F applied to an elastomer with thickness *h*. Pressure distribution along the intersection between the undersurface and cross-section (B) under varying external forces and (C) with varying elastomer thicknesses. (D) Pressure distribution on the undersurface of a 5 mm-thickness elastomer under a 5 N force.

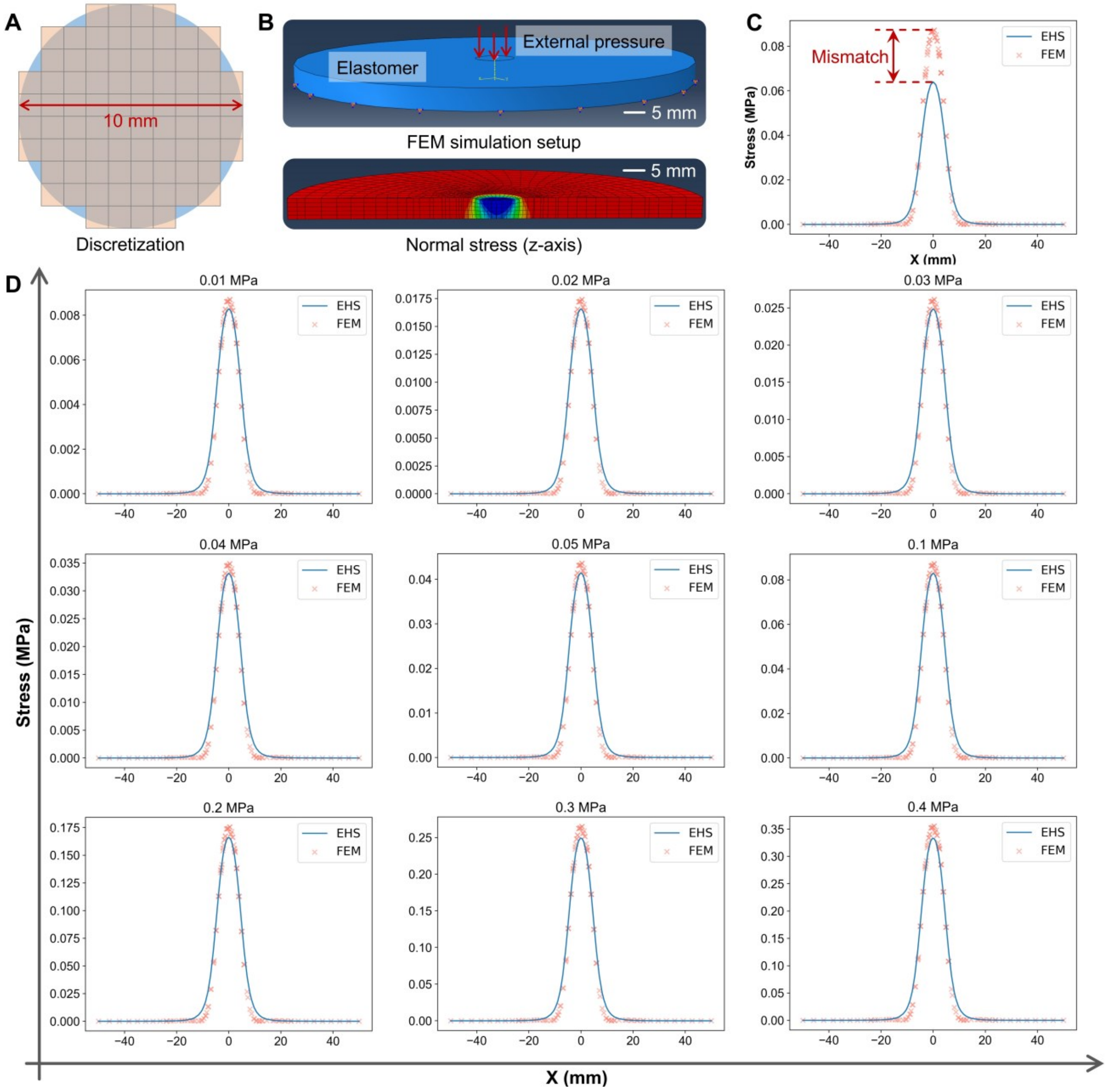

Fig. S15. Generation of ground-truth pressure distribution. (A) Discretization of the surface contact into 80 point contacts. (B) Setup and results of the FEM simulation. (C) The mismatch between FEM simulation and EHS results. (D) Corrected results after applying adjustments.

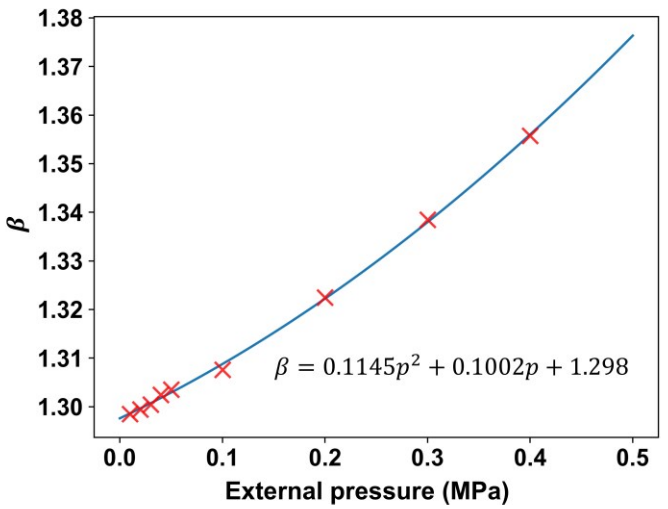

Fig. S16. Relationship between the correction factor $\boldsymbol{\beta}$ and external pressure ($\boldsymbol{p}$).

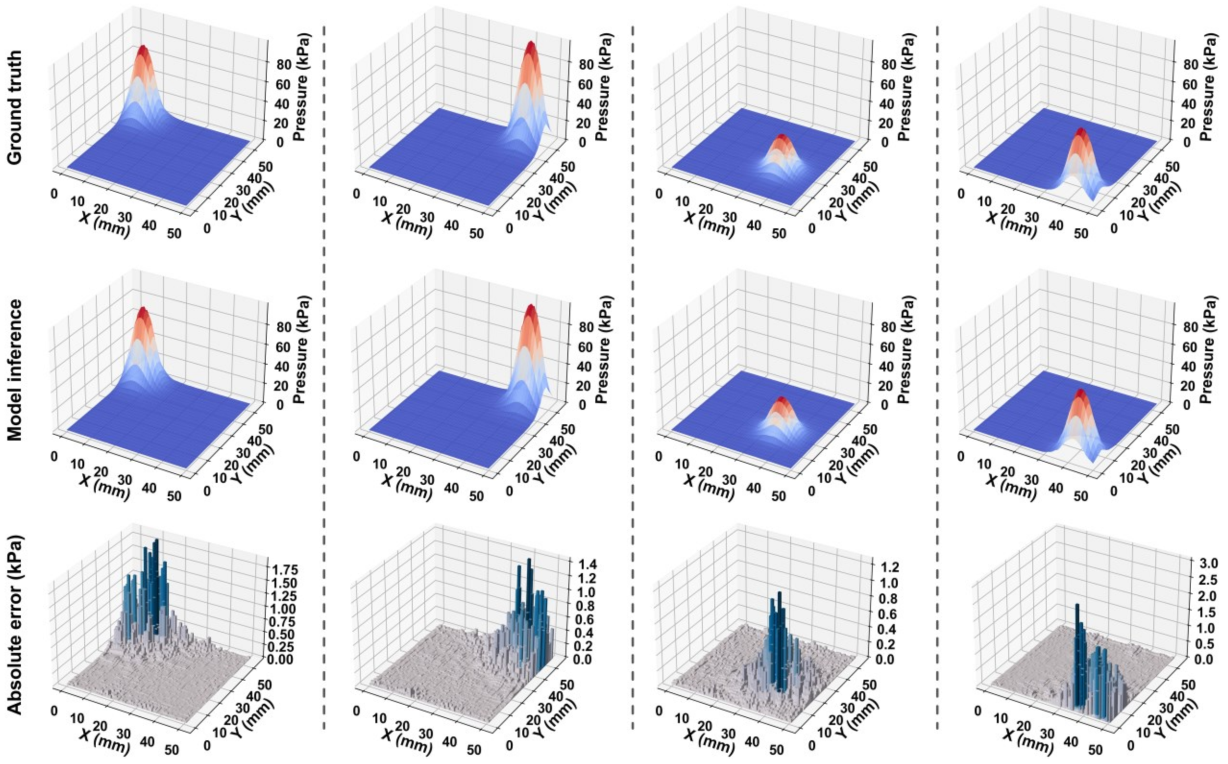

Fig. S17. Performance of the SATS model's inference on pressure distribution. The third row shows the absolute pressure error at each position across the sensing surface.

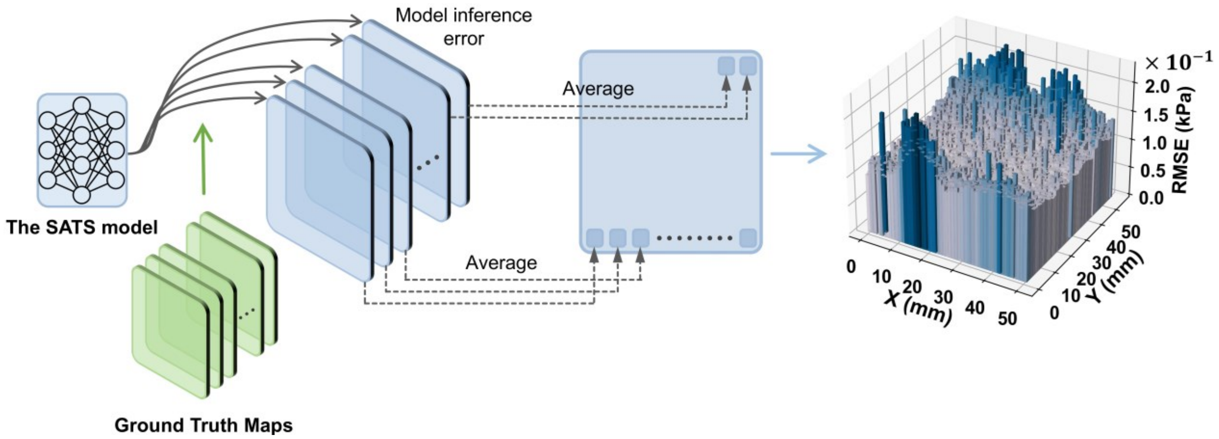

Fig. S18. Schematic diagram of error map calculation.

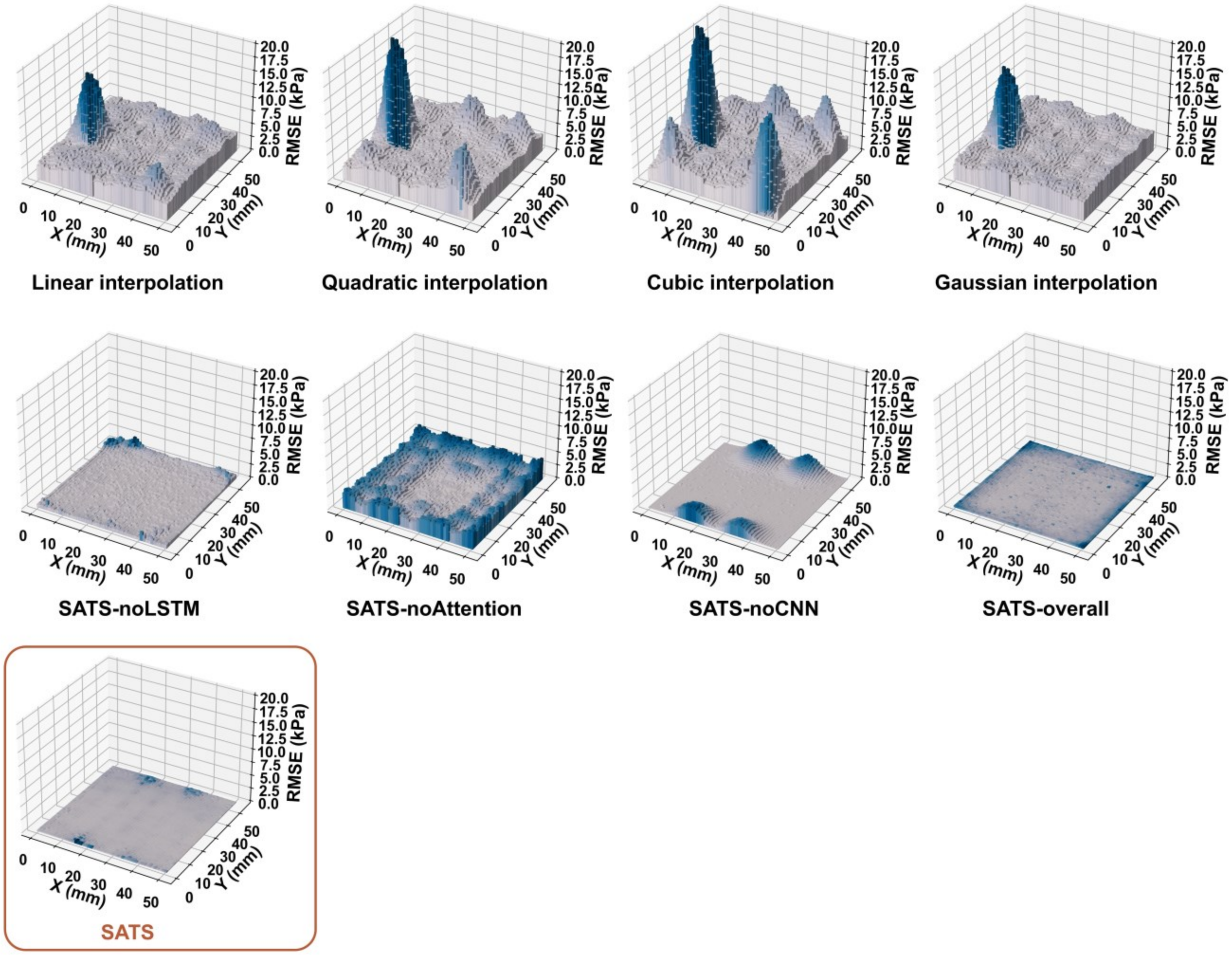

Fig. S19. Error distributions, with respect to the pressing position, of different interpolation methods and variants of the SATS model.

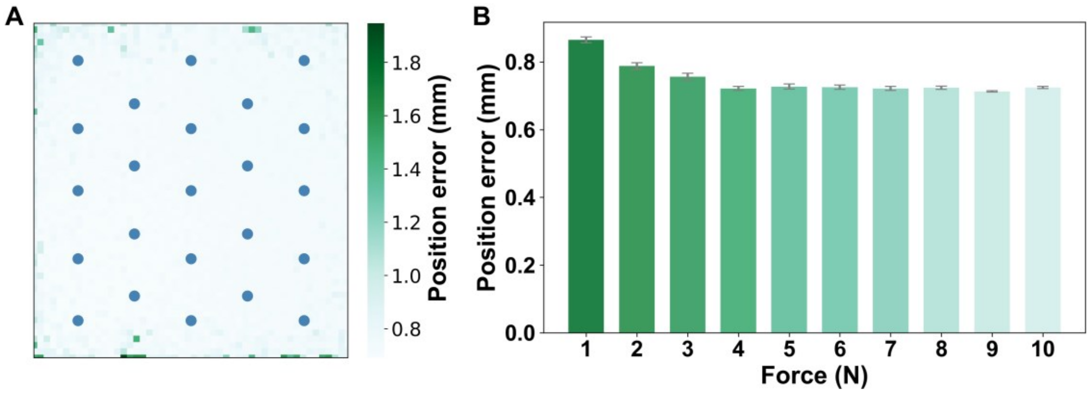

Fig. S20. Localization error in relation to (A) pressing position and (B) external force. Error bars indicate the standard error of the mean.

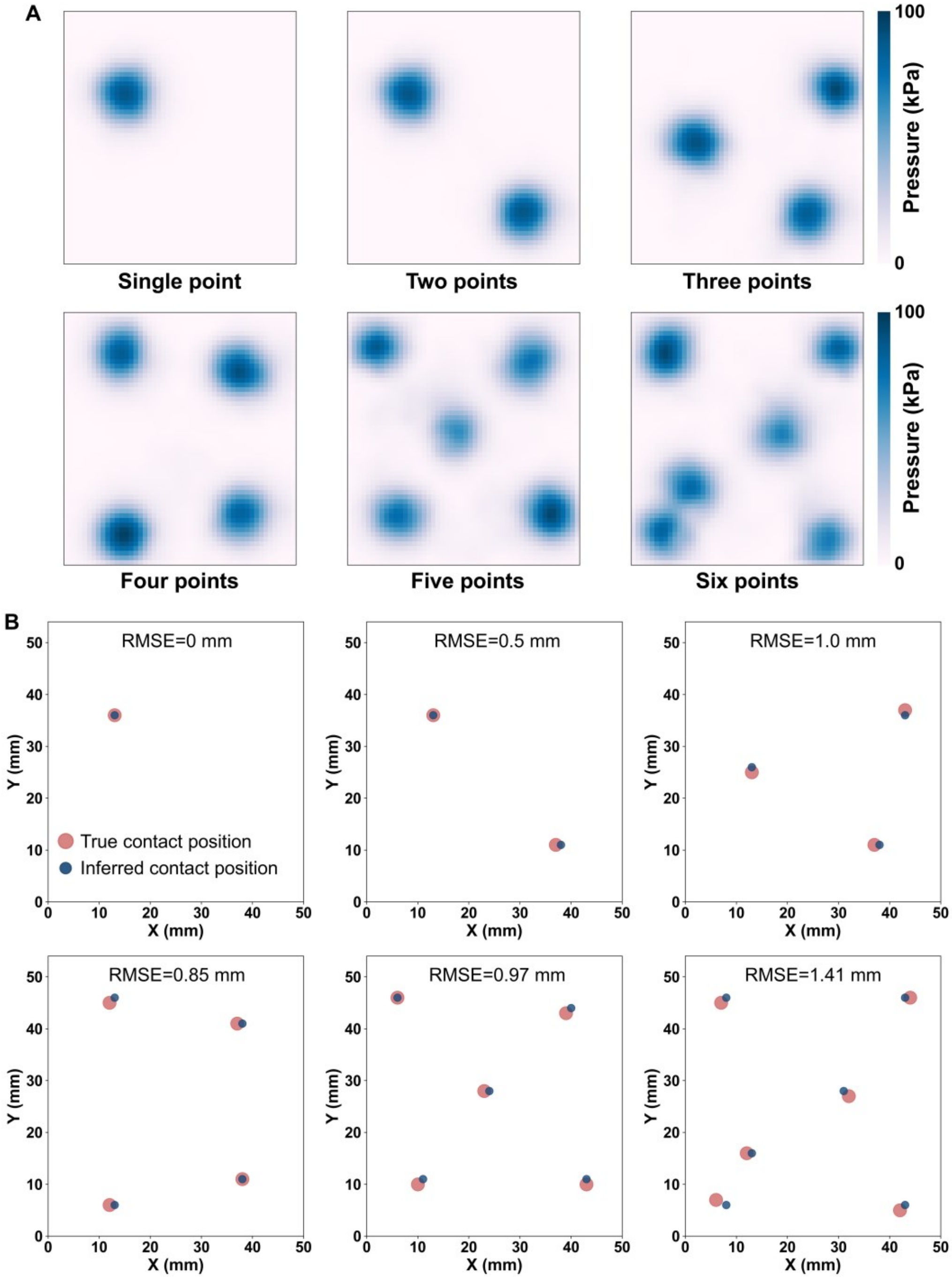

Fig. S21. More examples of multi-point contact detection, including simultaneously detecting one, two, three, four, five, and six points. (A) Inferred pressure distributions. (B) Correspondence between true contact positions and inferred contact positions (determined by the NMS algorithm based on the pressure maps).

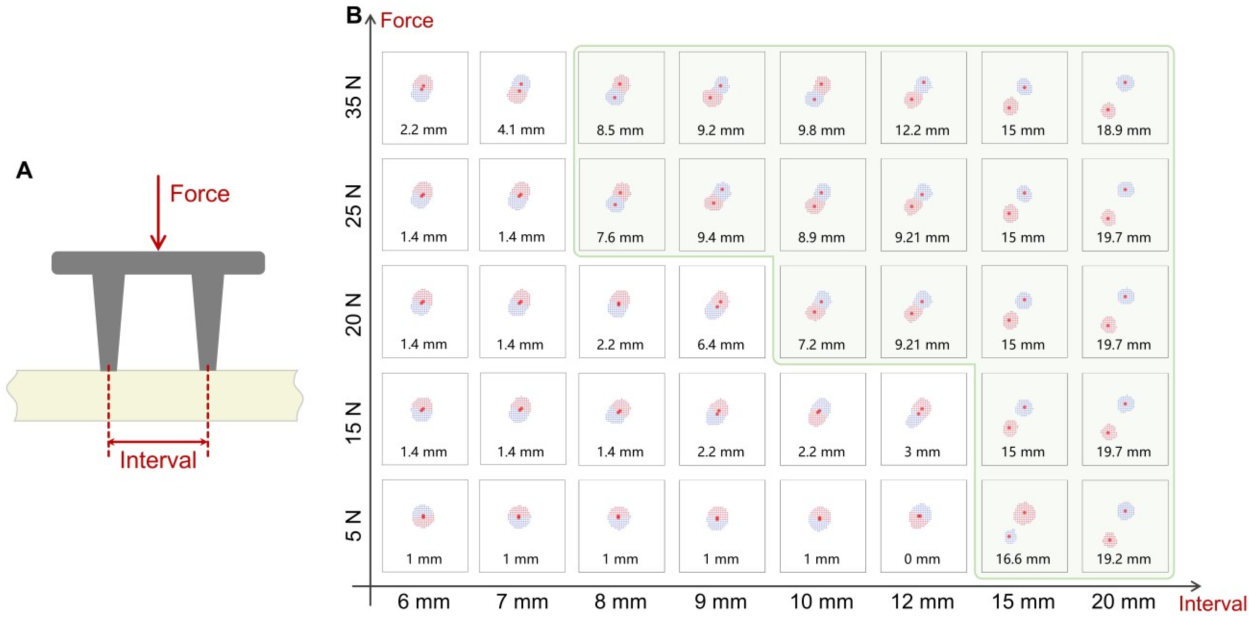

Fig. S22. Two-point discrimination experiment. (A) Experimental setup. (B) Results of discrimination under varying forces and different intervals. Insets with green backgrounds can be successfully distinguished. Numerical values in the subplots indicate the detected interval between two contact positions.

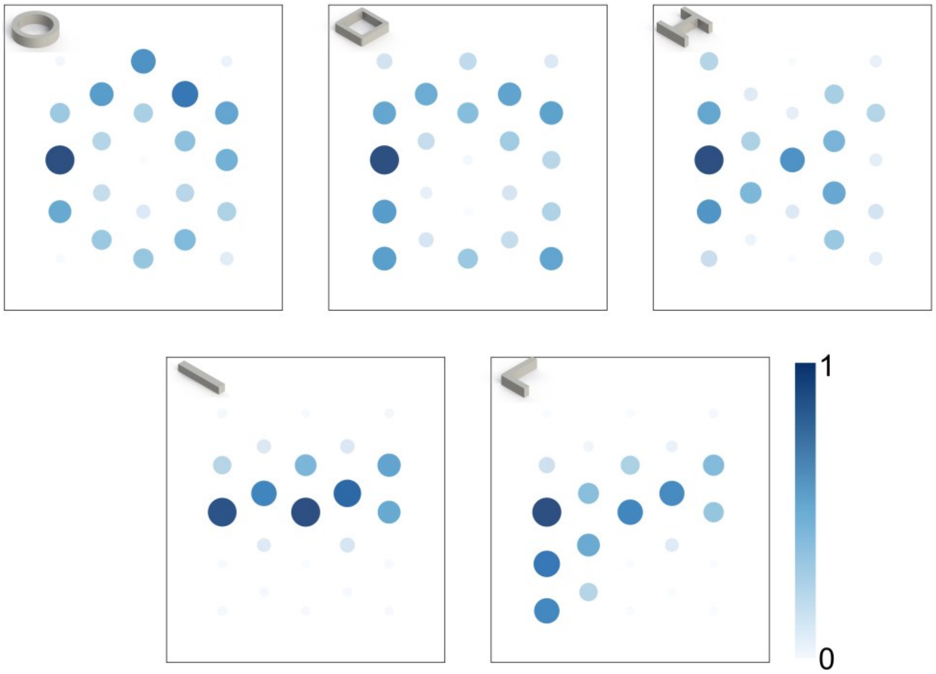

Fig. S23. Normalized activations of sensing units subjected to pressure from five shapes. Darker colors indicate stronger responses.

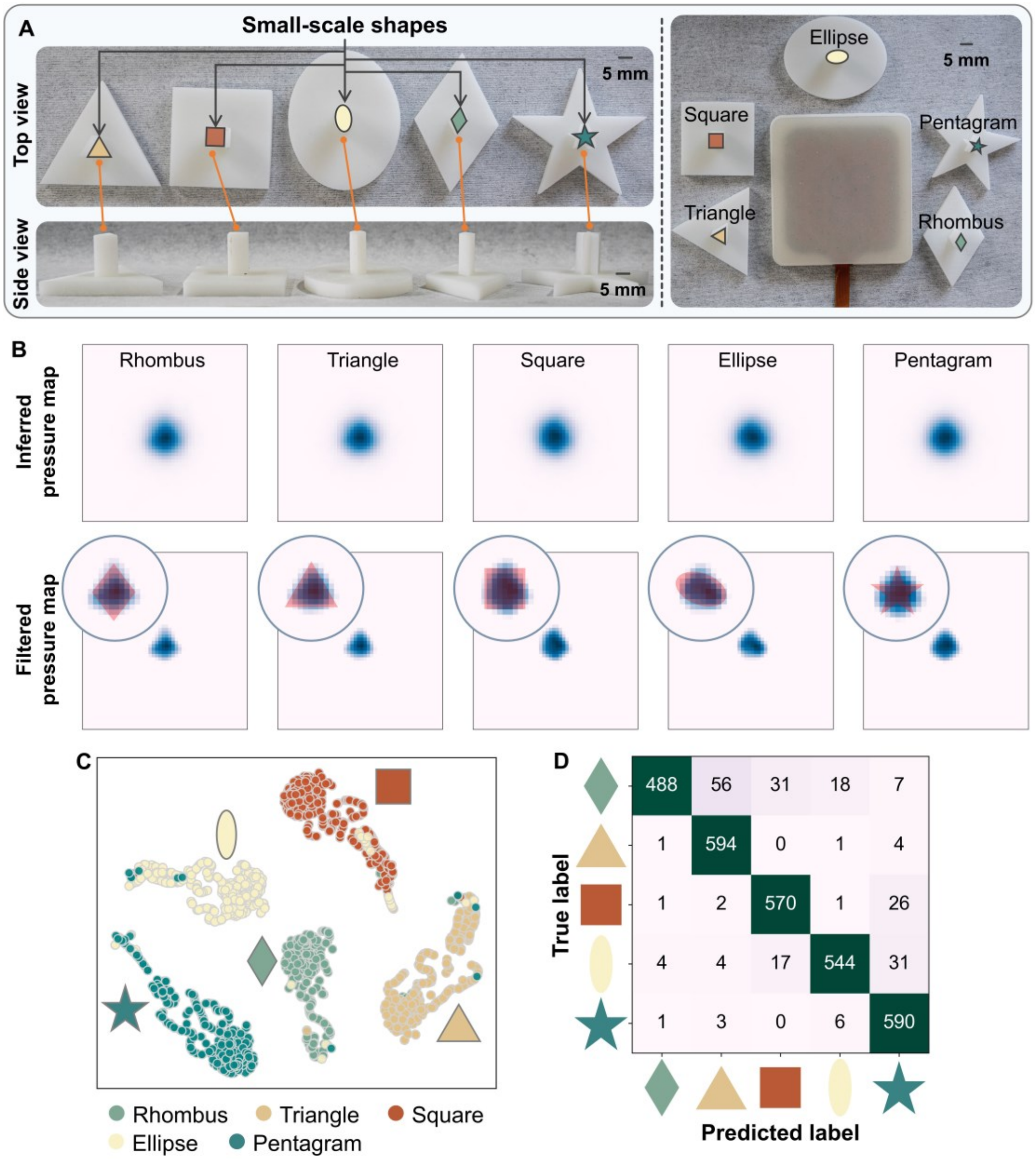

Fig. S24. Recognition of small-scale shapes. (A) Five small-scale shapes to recognize. (B) The original pressure maps inferred by the SATS model (first row), and the corresponding pressure maps after applying Laplace and Gaussian kernels (second row). (C) Dimension reduction of the test data using the t-SNE algorithm. (D) Confusion matrix for the classification of different shapes.

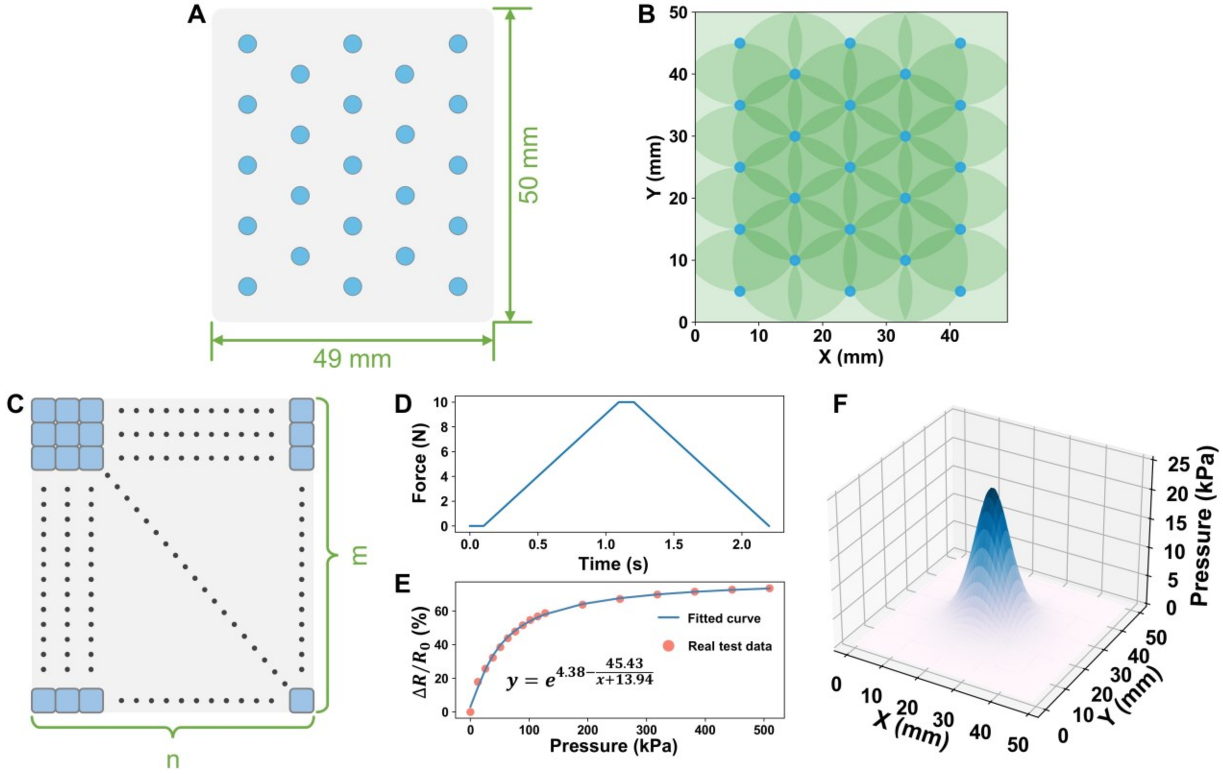

Fig. S25. Illustration of the simulated sensing array. (A) The boundary dimensions of the sensing surface. (B) The overall receptive field of the sensing array. (C) The method for partitioning the sensing surface for data collection, dividing the entire surface into an m×n grid. (D) The simulated loading process at each position. (E) The simulated sensor response to applied pressure referring to real responses. (F) Pressure distribution across the sensing surface under an external force of 5 N at location [25 mm, 25 mm].

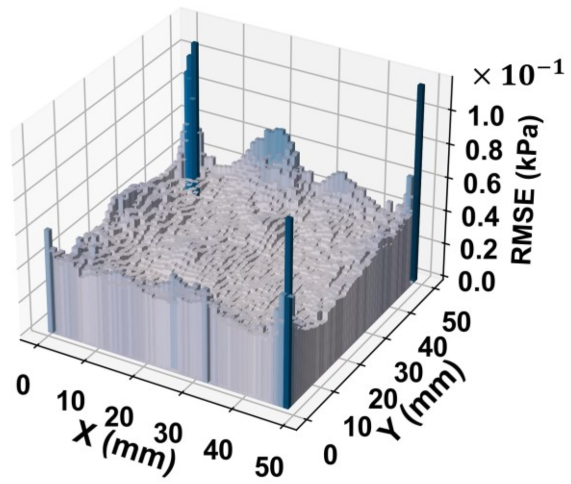

Fig. S26. Error distribution in relation to the pressing position, as obtained from training the SATS model with simulated data.

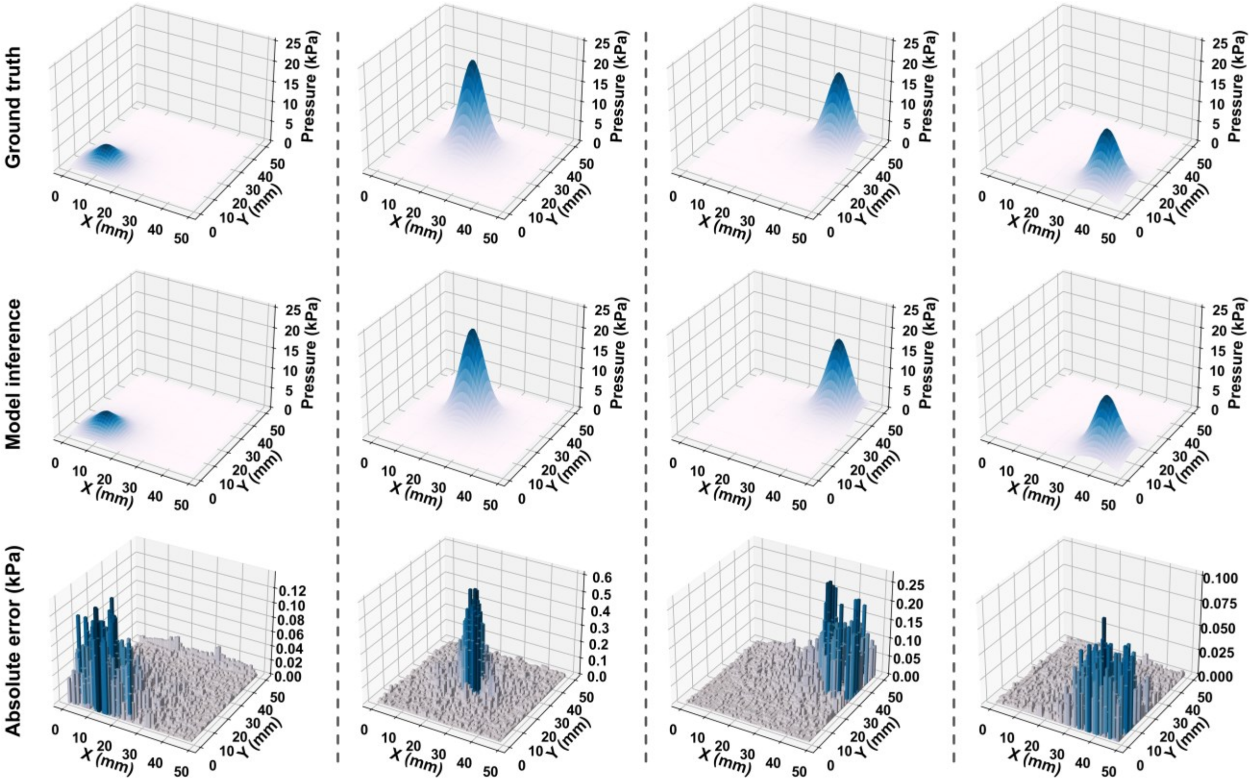

Fig. S27. Single-point contact pressure distributions inferred by the SATS model trained on simulated data.

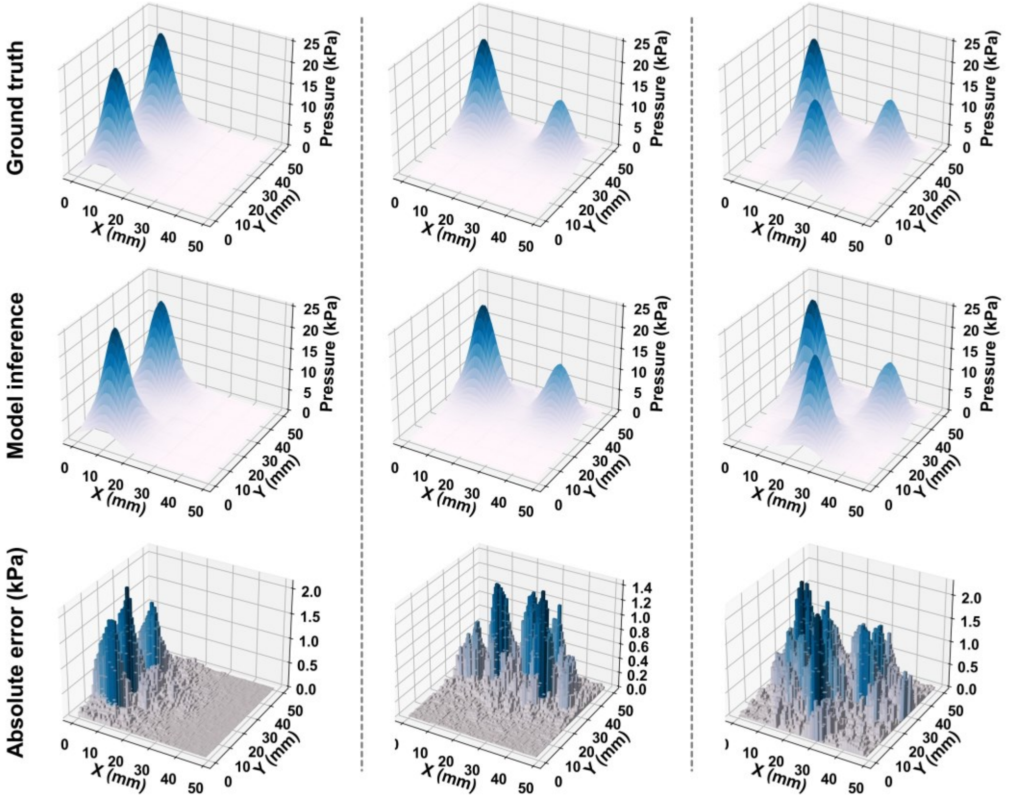

Fig. S28. Multi-point contact pressure distributions inferred by the SATS model trained on simulated data.

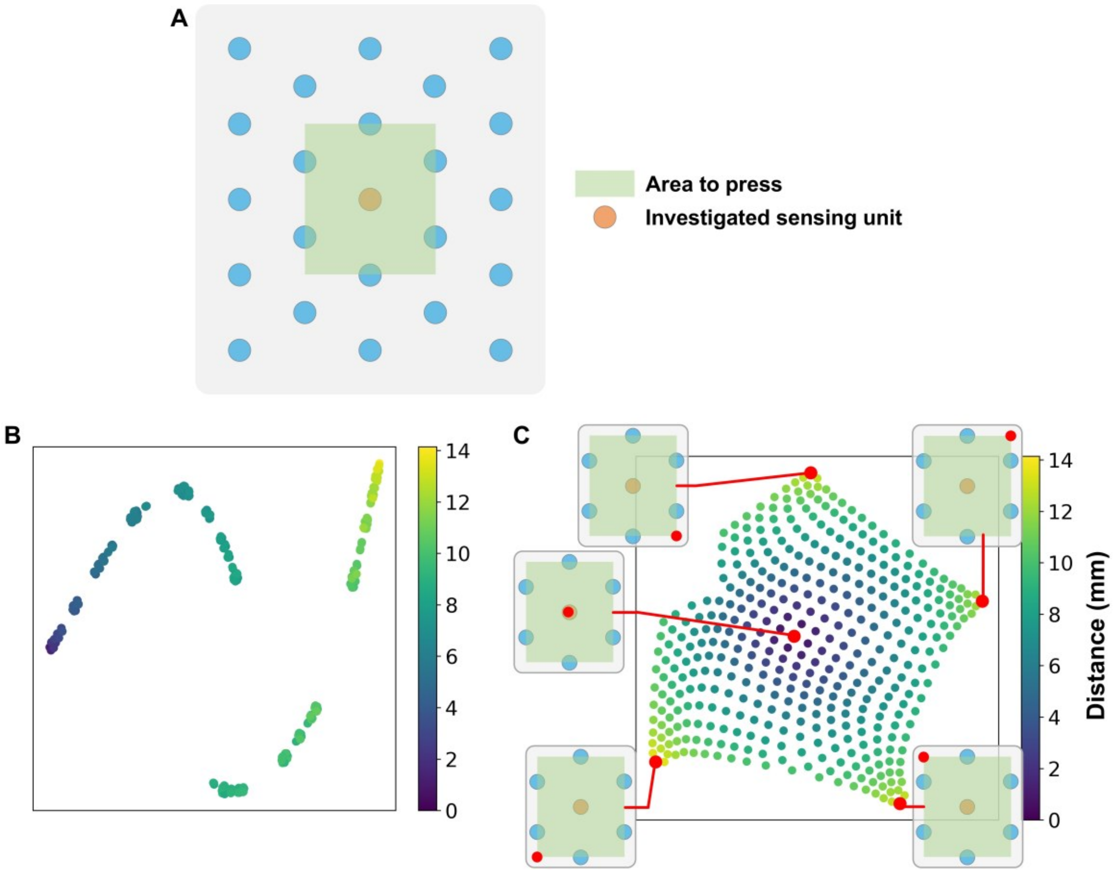

Fig. S29. Investigation into the SATS model's interpretability. (A) Illustration of the pressing area and the investigated sensing unit. (B) Dimension-reduced features encoded by the LSTM layer. (C) Dimension-reduced features encoded by the self-attention module and the correspondence between pressing positions and features.

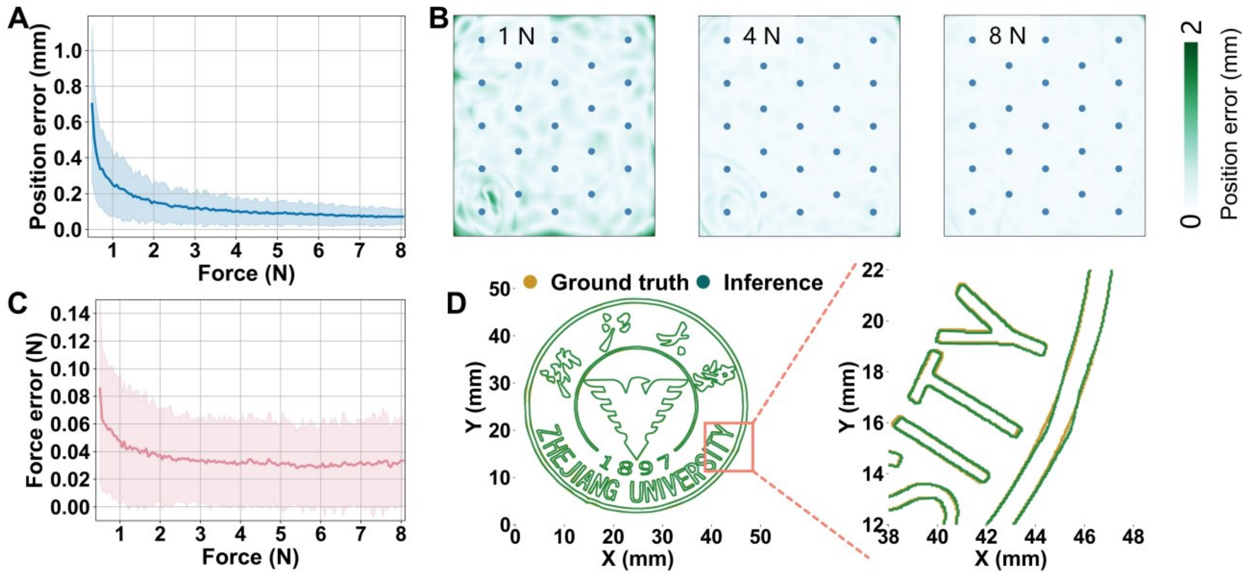

Fig. S30. Performance of the SATS model's coordinate and force inference capabilities. (A) Position error (RMSE) relative to the external force. (B) Position error (RMSE) relative to the pressing position under varying forces. (C) Force inference error relative to the external force. (D) Results of a contour-following application, where an external force of 8 N was simulated to press each point, yielding an average position error of 0.061 mm.

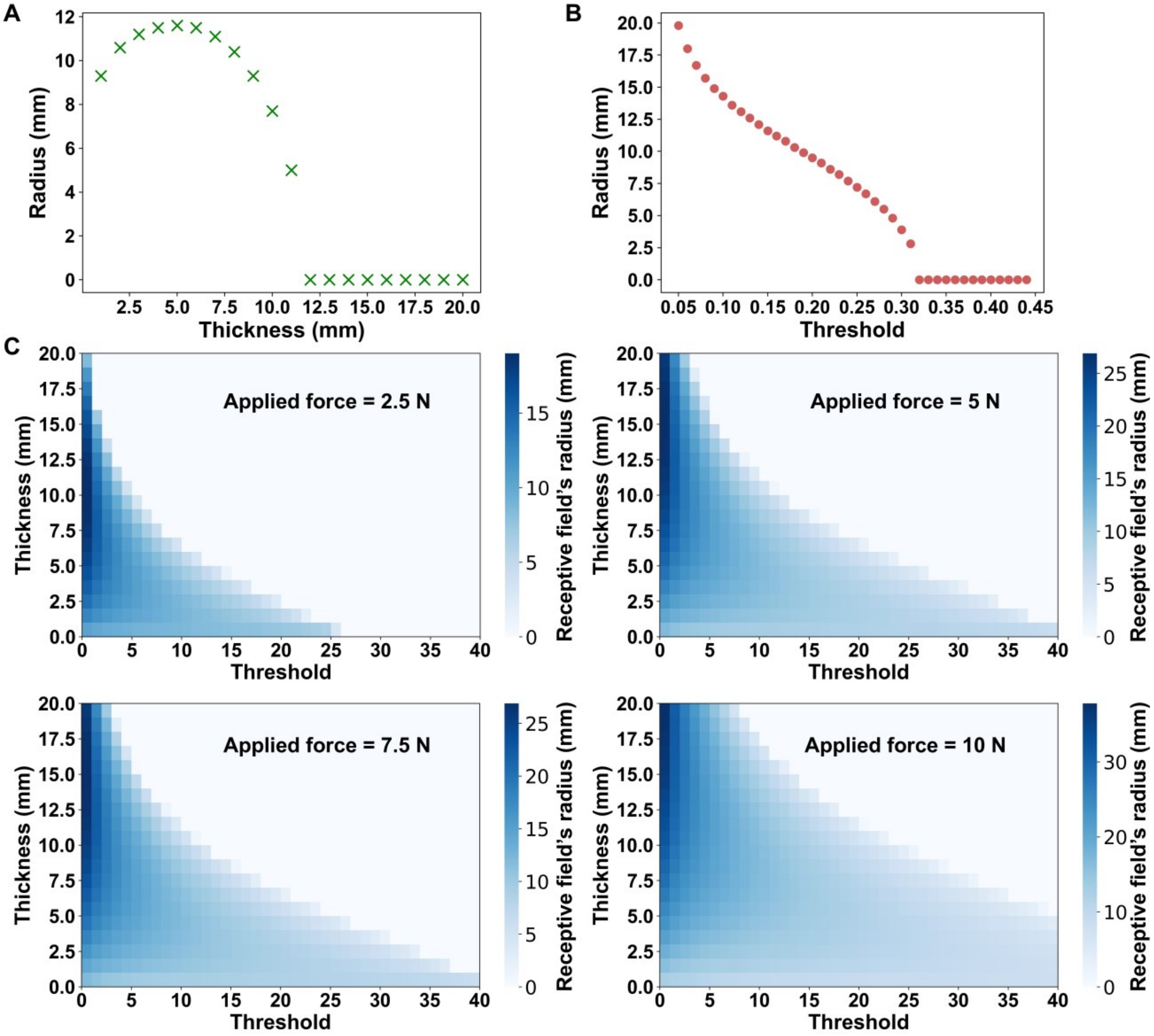

Fig. S31. Investigation of the optimal receptive field. Relationship between the receptive field radius and (A) the thickness of the elastic covering and (B) the sensing unit's detection threshold. (C) Combined effects of thickness, threshold, and applied force magnitude on the receptive field radius.

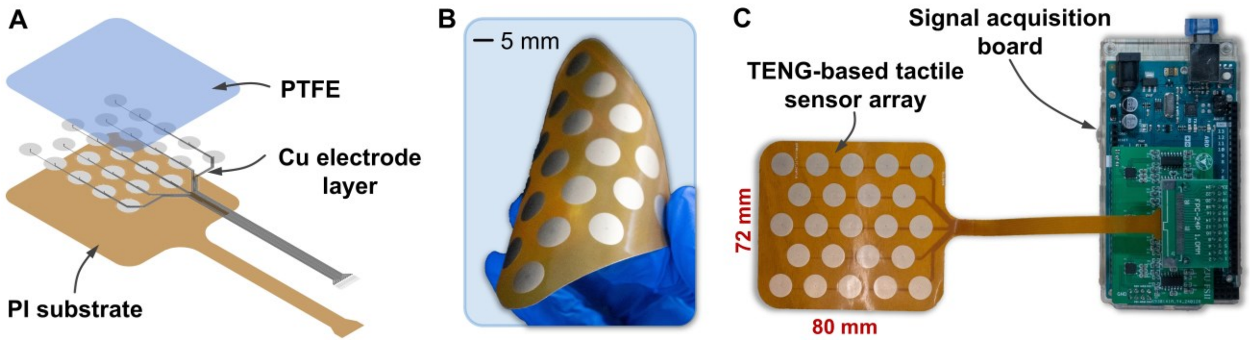

Fig. S32. The TENG-based tactile sensor for dynamic stimuli perception. (A) Explosive view of the TENG tactile sensor, manufactured as a flexible printed circuit board. PTFE indicates Polytetrafluoroethylene and PI indicates Polyimide. (B) Illustration on the flexibility of the TENG sensor. (C) Photograph of the integrated sensor system comprising the TENG sensor and a signal acquisition circuit board.

Table S1. Comparison of state-of-the-art multi-point SR solutions and our design.

References	Sensing mechanism	Sensing area	Localization error	No. of physical and virtual taxels	SR scale factor
**[36]**	Resistive	40000 mm^2	4.2 mm	32; ~722	~23
**[37]**	Resistive	40000 mm^2	4.88 mm	7; ~535	~76
**[38]**	Not available	24000 mm^2	5 mm	10; 240	24
**[39]**	Magnetic	~400 mm^2	-	16; 1600	100
**[40]**	Resistive	3600 mm^2	-	25; 400	16
**This work**	Resistive	2700 mm^2	**0.73 mm**	**23; 2700**	**~117**

Table S2. Comparison of the SATS model with other methods.

Method	Average RMSE error (kPa)		
	**Training set**	**Test set**	**Union set**
Linear interpolation	4.265	4.248	4.262
Quadric interpolation	4.598	4.582	4.595
Cubic interpolation	5.387	5.367	5.383
Gaussian interpolation	3.948	3.930	3.945
SATS-noLSTM	0.432	0.437	0.433
SATS-noAttention	1.755	1.808	1.763
SATS-noCNN	0.414	0.429	0.417
SATS-overall	0.186	0.197	0.187
SATS (original version)	**0.113**	**0.130**	**0.116**

Movie S1.
Demonstration of Multi-Point Tactile Super-Resolution using the MSR-Skin

Movie S2.
Customizing a Miniaturized Keyboard onto the MSR-Skin Based on High-Precision Tactile Perception

Movie S3.
The Miniaturized Keyboard's Application as the Input Device of a calculator

Movie S4.
Covering a Robot Hand with the MSR-Skin to Provide Embodied Perception

Movie S5.
Online Classification of Various Shapes Using the Proposed MSR-Skin

Movie S6.
The TENG-Based Sensor for Dynamic Perception with Enhanced Spatial Resolution

REFERENCES AND NOTES

1. R. Chrisley, Embodied artificial intelligence. *Artif Intell* **149**, 131–150 (2003).

2. R. Dahiya, E-skin: From humanoids to humans. *Proc. IEEE* **107**, 247–252 (2019).

3. Y. Liu, W. Chen, Y. Bai, J. Luo, X. Song, K. Jiang, Z. Li, G. Zhao, J. Lin, G. Li, Aligning cyber space with physical world: A comprehensive survey on embodied AI. arXiv:2407.06886 [cs.CV] (2024).

4. H. Liu, D. Guo, F. Sun, W. Yang, S. Furber, T. Sun, Embodied tactile perception and learning. *Brain Sci. Adv.* **6**, 132–158 (2020).

5. N. Bai, Y. Xue, S. Chen, L. Shi, J. Shi, Y. Zhang, X. Hou, Y. Cheng, K. Huang, W. Wang, J. Zhang, Y. Liu, C. F. Guo, A robotic sensory system with high spatiotemporal resolution for texture recognition. *Nat. Commun.* **14**, 7121 (2023).

6. H. Qiao, S. Sun, P. Wu, Non-equilibrium-growing aesthetic ionic skin for fingertip-like strain-undisturbed tactile sensation and texture recognition. *Adv. Mater.* **35**, 2300593 (2023).

7. Z. Ye, G. Pang, K. Xu, Z. Hou, H. Lv, Y. Shen, G. Yang, Soft robot skin with conformal adaptability for on-body tactile perception of collaborative robots. *IEEE Robot. Autom. Lett.* **7**, 5127–5134 (2022).

8. Z. Liu, X. Hu, R. Bo, Y. Yang, X. Cheng, W. Pang, Q. Liu, Y. Wang, S. Wang, S. Xu, Z. Shen, Y. Zhang, A three-dimensionally architected electronic skin mimicking human mechanosensation. *Science* **384**, 987–994 (2024).

9. Q. Ouyang, C. Yao, H. Chen, L. Song, T. Zhang, D. Chen, L. Yang, M. Chen, H.-j. Chen, Z. Peng, X. Xie, Machine learning-coupled tactile recognition with high spatiotemporal resolution based on cross-striped nanocarbon piezoresistive sensor array. *Biosens. Bioelectron.* **246**, 115873 (2024).

10. S. B. Choi, T. Noh, S. B. Jung, J. W. Kim, Stretchable piezoresistive pressure sensor array with sophisticated sensitivity, strain-insensitivity, and reproducibility. *Adv. Sci.* **11**, e2405374 (2024).

11. Q. Su, Q. Zou, Y. Li, Y. Chen, S.-Y. Teng, J. T. Kelleher, R. Nith, P. Cheng, N. Li, W. Liu, A stretchable and strain-unperturbed pressure sensor for motion interference–free tactile monitoring on skins. *Sci. Adv.* **7**, eabi4563 (2021).

12. Q. Yang, Z. Ye, R. Wu, H. Lv, C. Li, K. Xu, G. Yang, A highly sensitive iontronic bimodal sensor with pressure-temperature discriminability for robot skin. *Adv. Mater. Technol.* **8**, 2300561 (2023).

13. T. Wang, Y. Zhao, Q. Wang, A flexible iontronic capacitive sensing array for hand gesture recognition using deep convolutional neural networks. *Soft Robot.* **10**, 443–453 (2023).

14. Y. Lu, D. Kong, G. Yang, R. Wang, G. Pang, H. Luo, H. Yang, K. Xu, Machine learning-enabled tactile sensor design for dynamic touch decoding. *Adv. Sci.* **10**, e2303949 (2023).

15. P. Yang, Y. Shi, S. Li, X. Tao, Z. Liu, X. Wang, Z. L. Wang, X. Chen, Monitoring the degree of comfort of shoes in-motion using triboelectric pressure sensors with an ultrawide detection range. *ACS Nano* **16**, 4654–4665 (2022).

16. Z. Yan, L. Wang, Y. Xia, R. Qiu, W. Liu, M. Wu, Y. Zhu, S. Zhu, C. Jia, M. Zhu, R. Cao, Z. Li, X. Wang, Flexible high-resolution triboelectric sensor array based on patterned laser-induced graphene for self-powered real-time tactile sensing. *Adv. Funct. Mater.* **31**, 2100709 (2021).

17. Y. Yan, Z. Hu, Z. Yang, W. Yuan, C. Song, J. Pan, Y. Shen, Soft magnetic skin for super-resolution tactile sensing with force self-decoupling. *Sci. Robot.* **6**, eabc8801 (2021).

18. J. Man, Z. Jin, J. Chen, Magnetic tactile sensor with bionic hair array for sliding sensing and object recognition. *Adv. Sci.* **11**, e2306832 (2024).

19. W. Yuan, S. Dong, E. H. Adelson, GelSight: High-resolution robot tactile sensors for estimating geometry and force. *Sensors* **17**, 2762 (2017).

20. H. Sun, K. J. Kuchenbecker, G. Martius, A soft thumb-sized vision-based sensor with accurate all-round force perception. *Nat. Mach. Intell.* **4**, 135–145 (2022).

21. V. E. Abraira, D. D. Ginty, The sensory neurons of touch. *Neuron* **79**, 618–639 (2013).

22. M. L. Hammock, A. Chortos, B. C. K. Tee, J. B. H. Tok, Z. Bao, 25th anniversary article: The evolution of electronic skin (E-skin): A brief history, design considerations, and recent progress. *Adv. Mater.* **25**, 5997–6038 (2013).

23. K. O. Johnson, The roles and functions of cutaneous mechanoreceptors. *Curr. Opin. Neurobiol.* **11**, 455–461 (2001).

24. R. S. Johansson, J. R. Flanagan, Coding and use of tactile signals from the fingertips in object manipulation tasks. *Nat. Rev. Neurosci.* **10**, 345–359 (2009).

25. J. M. Loomis, An investigation of tactile hyperacuity. *Sens. Processes* **3**, 289–302 (1979).

26. J. M. Loomis, C. C. Collins, Sensitivity to shifts of a point stimulus: An instance of tactile hyperacuity. *Percept. Psychophys.* **24**, 487–492 (1978).

27. N. F. Lepora, U. Martinez-Hernandez, M. Evans, L. Natale, G. Metta, T. J. Prescott, Tactile superresolution and biomimetic hyperacuity. *IEEE Trans. Robot.* **31**, 605–618 (2015).

28. K. R. Pyun, K. Kwon, M. J. Yoo, K. K. Kim, D. Gong, W.-H. Yeo, S. Han, S. H. Ko, Machine-learned wearable sensors for real-time hand-motion recognition: Toward practical applications. *Natl. Sci. Rev.* **11**, nwad298 (2024).

29. K. K. Kim, I. Ha, M. Kim, J. Choi, P. Won, S. Jo, S. H. Ko, A deep-learned skin sensor decoding the epicentral human motions. *Nat. Commun.* **11**, 2149 (2020).

30. K. K. Kim, M. Kim, K. Pyun, J. Kim, J. Min, S. Koh, S. E. Root, J. Kim, B.-N. T. Nguyen, Y. Nishio, S. Han, J. Choi, C. Y. Kim, J. B. H. Tok, S. Jo, S. H. Ko, Z. Bao, A substrate-less nanomesh receptor with meta-learning for rapid hand task recognition. *Nat. Electron.* **6**, 64–75 (2022).

31. X. Li, Y. Zhang, X. Xie, J. Li, G. Shi, in *Proceedings of the AAAI Conference on Artificial Intelligence* (AAAI Press, 2023), vol. 37, pp. 6192–6199.

32. L. Massari, G. Fransvea, J. D'Abbraccio, M. Filosa, G. Terruso, A. Aliperta, G. D'Alesio, M. Zaltieri, E. Schena, E. Palermo, E. Sinibaldi, C. M. Oddo, Functional mimicry of Ruffini receptors with fibre Bragg gratings and deep neural networks enables a bio-inspired large-area tactile-sensitive skin. *Nat. Mach. Intel.* **4**, 425–435 (2022).

33. M.-Y. Cho, J.-W. Lee, C. Park, B. D. Lee, J. S. Kyeong, E. J. Park, K. Y. Lee, K.-S. Sohn, Large-area piezoresistive tactile sensor developed by training a super-simple single-layer carbon nanotube-dispersed polydimethylsiloxane pad. *Adv. Intell. Syst.* **4**, 2100123 (2021).

34. H. Sun, G. Martius, Guiding the design of superresolution tactile skins with taxel value isolines theory. *Sci. Robot.* **7**, eabm0608 (2022).

35. P. Piacenza, S. Sherman, M. Ciocarlie, Data-driven super-resolution on a tactile dome. *IEEE Robot. Autom. Lett.* **3**, 1434–1441 (2018).

36. K. Park, H. Yuk, M. Yang, J. Cho, H. Lee, J. Kim, A biomimetic elastomeric robot skin using electrical impedance and acoustic tomography for tactile sensing. *Sci. Robot.* **7**, eabm7187 (2022).

37. H. Park, K. Park, S. Mo, J. Kim, Deep neural network based electrical impedance tomographic sensing methodology for large-area robotic tactile sensing. *IEEE Trans. Robot.* **37**, 1570–1583 (2021).

38. H. Sun, G. Martius, Machine learning for haptics: Inferring multi-contact stimulation from sparse sensor configuration. *Front. Neurorobot.* **13**, 51 (2019).

39. B. Wu, Q. Liu, Q. Zhang, in *2022 IEEE/RSJ International Conference on Intelligent Robots and Systems (IROS)* (IEEE, 2022), pp. 3644–3650.

40. M. Kim, H. Choi, K.-J. Cho, S. Jo, Single to multi: Data-driven high resolution calibration method for piezoresistive sensor array. *IEEE Robot. Autom. Lett.* **6**, 4970–4977 (2021).

41. D. Kong, G. Yang, G. Pang, Z. Ye, H. Lv, Z. Yu, F. Wang, X. V. Wang, K. Xu, H. Yang, Bioinspired co-design of tactile sensor and deep learning algorithm for human-robot interaction. *Adv. Intell. Syst.* **4**, 2200050 (2022).

42. H. Hu, C. Zhang, X. Lai, H. Dai, C. Pan, H. Sun, D. Tang, Z. Hu, J. Fu, T. Li, P. Zhao, Large-area magnetic skin for multi-point and multi-scale tactile sensing with super-resolution. *npj Flex Electron.* **8**, 42 (2024).

43. H. Hu, C. Zhang, C. Pan, H. Dai, H. Sun, Y. Pan, X. Lai, C. Lyu, D. Tang, J. Fu, P. Zhao, Wireless flexible magnetic tactile sensor with super-resolution in large-areas. *ACS Nano* **16**, 19271–19280 (2022).

44. C. Pasluosta, P. Kiele, T. Stieglitz, Paradigms for restoration of somatosensory feedback via stimulation of the peripheral nervous system. *Clin. Neurophysiol.* **129**, 851–862 (2018).

45. G. Strang, *Introduction to Linear Algebra* (Wellesley-Cambridge Press, 2022).

46. M.-Y. Cho, J. H. Lee, S.-H. Kim, J. S. Kim, S. Timilsina, An extremely inexpensive, simple, and flexible carbon fiber electrode for tunable elastomeric piezo-resistive sensors and devices realized by LSTM RNN. *ACS Appl. Mater. Interfaces* **11**, 11910–11919 (2019).

47. X. Li, X. Hu, X. Chen, J. Fan, Z. Zhao, J. Wu, H. Wang, Q. Dai, Spatial redundancy transformer for self-supervised fluorescence image denoising. *Nat. Comput. Sci.* **3**, 1067–1080 (2023).

48. W. M. Kouw, M. Loog, An introduction to domain adaptation and transfer learning. arXiv:1812.11806 [cs.LG] (2018).

49. H. Lee, H. Sun, H. Park, G. Serhat, B. Javot, G. Martius, K. J. Kuchenbecker, Predicting the force map of an ERT-based tactile sensor using simulation and deep networks. *IEEE Trans Autom Sci Eng* **20**, 425–439 (2023).

50. K. L. Johnson, *Contact Mechanics* (Cambridge Univ. Press, 1987).

51. Q. Diao, W. Chen, Y. Wang, Q. Jiang, Z. Li, in *International Conference on Intelligent Robotics and Applications* (Springer, 2022), chap. 59, pp. 652–660.

52. W. Liu, C. Gu, R. Zeng, P. Yu, X. Fu, A novel inverse solution of contact force based on a sparse tactile sensor array. *Sensors* **18**, 351 (2018).

53. H. Yokota, N. Otsuru, R. Kikuchi, R. Suzuki, S. Kojima, K. Saito, S. Miyaguchi, Y. Inukai, H. Onishi, Establishment of optimal two-point discrimination test method and consideration of reproducibility. *Neurosci. Lett.* **714**, 134525 (2020).

54. P. Veliˇckovi'c, G. Cucurull, A. Casanova, A. Romero, P. Li'o, Y. Bengio, Graph attention networks. arXiv:1710.10903 [stat.ML] (2017).

55. B. Xu, N. Wang, T. Chen, M. Li, Empirical evaluation of rectified activations in convolutional network. arXiv:1505.00853 [cs.LG] (2015).

56. E. Jang, S. Gu, B. Poole, Categorical reparameterization with gumbel-softmax. arXiv:1611.01144v5 [stat.ML] (2016).

57. Clevert, Djork-Arné, Unterthiner, Thomas, Hochreiter, S. Fast and accurate deep network learning by exponential linear units (ELUs). 2016.

58. G. Vásárhelyi, "The design of tactile sensors and their elastic cover," thesis, Péter Pázmány Catholic University (2007).
